# Mapping the IscR regulon sheds light on the regulation of iron homeostasis in *Caulobacter*

**DOI:** 10.3389/fmicb.2024.1463854

**Published:** 2024-09-30

**Authors:** Naara M. dos Santos, Beatriz A. Picinato, Lucas S. Santos, Hugo L. de Araújo, Andrea Balan, Tie Koide, Marilis V. Marques

**Affiliations:** ^1^Departamento de Microbiologia, Instituto de Ciências Biomédicas, Universidade de São Paulo, São Paulo, Brazil; ^2^Departamento de Bioquímica e Imunologia, Faculdade de Medicina de Ribeirão Preto, Universidade de São Paulo, Ribeirão Preto, Brazil

**Keywords:** iron–sulfur cluster, IscR, Fur, gene regulation, *Caulobacter crescentus*

## Abstract

The role of the iron–sulfur [Fe-S] cluster transcriptional regulator IscR in maintaining [Fe-S] homeostasis in bacteria is still poorly characterized in many groups. *Caulobacter crescentus* and other Alphaproteobacteria have a single operon encoding [Fe-S] cluster biosynthesis enzymes. We showed that the expression of this operon increases in iron starvation, but not in oxidative stress, and is controlled mainly by IscR. Transcriptome analysis comparing an *iscR* null mutant strain with the wild-type (wt) strain identified 94 differentially expressed genes (DEGs), with 47 upregulated and 47 downregulated genes in the Δ*iscR* mutant. We determined the IscR binding sites in conditions of sufficient or scarce iron by Chromatin Immunoprecipitation followed by DNA sequencing (ChIP-seq), identifying two distinct putative DNA binding motifs. The estimated IscR regulon comprises 302 genes, and direct binding to several regulatory regions was shown by Electrophoresis Mobility Shift Assay (EMSA). The results showed that the IscR and Fur regulons partially overlap and that IscR represses the expression of the respiration regulator FixK, fine-tuning gene regulation in response to iron and redox balance.

## Introduction

Iron is an essential trace nutrient, necessary for several enzymes that use it as a cofactor. It can bind to proteins in the free Fe^2+^ form, or in the prosthetic groups heme and iron–sulfur [Fe-S] cluster. The predominantly insoluble Fe^3+^ form can be bound to organic molecules (siderophores) for uptake by specific transporters (Kramer et al., 2020). Within the cells as Fe^2+^, iron is incorporated in proteins, used to synthesize heme and [Fe-S] clusters, or stored in large ferritin complexes (Andrews et al., 2003). Iron homeostasis is tightly controlled by redundant mechanisms to ensure that no excess of free iron will lead to oxidative stress via the Fenton reaction, since [Fe-S] clusters are the main susceptible targets to reactive oxygen species (Imlay, 2006).

Bacterial iron homeostasis is mainly a result of transcriptional regulation mediated by Fur, a Fe^2+^-binding metalloprotein that represses the transcription of iron uptake genes and iron-using enzymes (Seo et al., 2014; Kramer et al., 2020). Fur may also act as a transcription activator, stimulating transcription of iron-using proteins under conditions of iron sufficiency, such as the NADH dehydrogenase (*nuoA-N* operon), aconitase (*acnA*), and succinate dehydrogenase (*sdhCBAD* operon) (Delany et al., 2004; da Silva Neto et al., 2009). In Enterobacteria, the activation of expression of Fur-regulated genes may also be indirect, where Fe^2+^-Fur represses the small regulatory RNA (sRNA) RyhB that inhibits gene expression at the post-transcriptional level (Massé and Gottesman, 2002). RyhB binds to its mRNA targets with the help of Hfq, inhibiting translation and triggering the degradation of both RNAs by the RNase E and the RNA degradosome (Massé et al., 2003; Desnoyers et al., 2009).

RyhB negatively regulates the mRNA of many [Fe-S]-using proteins (Prévost et al., 2011), including operons for [Fe-S] cluster biosynthesis (Desnoyers et al., 2009). Enterobacteria have two distinct operons that encode enzymes for the synthesis of [Fe-S] clusters, *iscRSUA-fdx-hscBA-fdx-iscX* and *sufABCDSE*, both containing all the proteins required for [Fe-S] cluster biosynthesis (Zheng et al., 1998; Takahashi and Tokumoto, 2002). Isc is the main housekeeping enzymatic system, responsible for [Fe-S] synthesis at normal conditions. When cells are under iron starvation or oxidative stress, there is a switch in gene expression leading to upregulation of the Suf system (Outten et al., 2004). This is advantageous because the scaffold protein SufB containing [2Fe-2S] clusters is not destabilized by hydrogen peroxide, while its equivalent IscU is sensitive to H_2_O (Ollagnier-De Choudens et al., 2003; Boyd et al., 2014; Remes et al., 2014; Pérard and Ollagnier de Choudens, 2018).

The increased expression of the *Escherichia coli* Suf system at low iron conditions is in part due to the exit of Fur from its operator at low iron conditions, but it is also positively regulated by the IscR regulator, encoded by the first gene in the *iscRSUA-fdx-hscBA-fdx-iscX* operon (Mettert and Kiley, 2014). IscR belongs to the transcription factor family Rrf2 (Mettert and Kiley, 2015), and in *E. coli* it regulates approximately 40 genes, including its own operon (Giel et al., 2006; Mettert and Kiley, 2015). IscR recognizes distinct operator sequences depending on whether it is in the holo (bound to a [2Fe-2S] group) or apo form; its own repression requires the binding of holo-IscR to one of the operator sequences in the *isc* promoter (Yeo et al., 2006; Fleischhacker et al., 2012). Besides transcriptional regulation, the *E. coli iscRSUA-fdx-hscBA-fdx-iscX* operon is also regulated by RyhB, which binds to a stem-loop structure in the *isc* mRNA upstream of the *iscS* gene. RyhB causes the degradation of the mRNA downstream of *iscR*, while the 5′ part remains stable and still produces functional apo-IscR that will in turn activate the expression of Suf (Desnoyers et al., 2009). The *suf* genes are also regulated by the oxidative stress regulator OxyR (Lee et al., 2004).

While there is a lot of detailed information about the regulation of Isc and Suf in *E. coli* and other Gammaproteobacteria, very little is known about it in other bacterial groups. Most Alphaproteobacteria, except for Rhizobiales and Rhodobacterales, have at least one Fur and one IscR ortholog (Johnston et al., 2007). The control of iron homeostasis in in the Alphaproteobacterium *Caulobacter crescentus* (also known as *Caulobacter vibrioides*) has been studied in previous works. We have characterized the Fur regulon and the response to iron starvation and identified iron transporters and storage proteins (da Silva Neto et al., 2009, 2013; de Castro Ferreira et al., 2016; Leaden et al., 2018). We have shown that when iron homeostasis is lost, there is an increase of oxidative stress, even at low iron conditions (Leaden et al., 2018), and that unlike *E. coli*, the catalase *katG* gene or any gene encoding oxidative stress detoxification enzymes are not regulated by Fur, suggesting that in *C. crescentus* there is an uncoupling of iron and H_2_O_2_-responsive regulations (Italiani et al., 2011; Silva et al., 2019).

In this work, we show that in *C. crescentus* there is only one operon encoding [Fe-S] cluster biosynthesis proteins, which contains genes orthologous to those belonging to both *E. coli isc* and *suf* operons. These aspects of iron metabolism that appear to be different from those described in enteric bacteria prompted us to investigate the regulatory role of IscR to determine its role in maintaining iron homeostasis in an Alphaproteobacterium. We have characterized the IscR regulon, both by global transcriptomic analysis of a null *iscR* mutant strain compared to wild-type (wt) and by Chromatin Immunoprecipitation followed by DNA sequencing (ChIP-seq). Some of the direct IscR targets were confirmed by Electrophoresis Mobility Shift Assay (EMSA) with an IscR-His expressed heterologously in *E. coli*. The IscR regulon comprises genes involved in oxidative stress response, oxygen concentration, transporters, riboflavin biosynthesis, basal energy metabolism and respiration under different oxygen levels, among others. Several IscR targets are also regulated by Fur, sometimes in opposite ways, indicating an interplay of these two regulators to modulate gene expression in response to the levels of Fe^2+^ and [Fe-S] clusters.

## Materials and methods

### Strains, growth conditions and plasmids

The *C. crescentus* strains used in this study were the wt strain NA1000 and isogenic mutant strains Δ*fur*, Δ*oxyR*, and Δ*iscR*; *E. coli* strains were DH10B, S17-1 and BL21(DE3) (described in Supplementary Table S1). When necessary, the plates were added of the following antibiotics: 1 μg/mL Tetracycline, 5 μg/mL Kanamycin, 20 μg/ml Nalidixic Acid (for *C. crescentus*) and 12.5 μg/mL Tetracycline, 50 μg/mL Kanamycin (for *E. coli*). Plasmids used in this work are described in Supplementary Table S2.

Eventually, after RNA sequencing, we realized that the wt NA1000 strain clone used for these experiments had spontaneously lost the genomic island corresponding to a mobile element (Marks et al., 2010) since no reads were obtained between positions 473,069–499,098. This clone was also used to construct the IscR-3xFLAG strain, so no reads were obtained for this region in the ChIP-seq analysis.

Bacterial cultures were grown under aerobic conditions at a temperature of 30°C in PYE rich culture medium (Ely, 1991) in flasks or in 96-well microplates (200 μL cultures). To investigate the effects of iron limitation in cultures, the iron chelator 2′,2-dipyridyl (DP) (Sigma-Aldrich) was added to a concentration of 100 μM during an incubation period of 2 h.

### Reciprocal best hits (RBH), synteny and protein structure analyses

*Escherichia coli* K12 MG1655 and *C. crescentus* NA1000 genomes were retrieved from NCBI databases. RBH analysis was conducted with easy-rbh workflow from MMseqs2 (v. 15.6f452) (Steinegger and Söding, 2017) and the results were analyzed by in-house Python scripts. Structural alignment of putative IscR proteins from selected bacteria was made with Expresso from webserver T-Coffee (Notredame et al., 2000). Gene synteny analysis was carried out with the with the SyntTax program (Oberto, 2013).

Protein structures were predicted using AlphaFold2 through the ColabFold platform (Mirdita et al., 2022). For each protein, 5 models were generated and ranked followed by built-in AMBER energy minimization of the top1 structure. Graphics were generated by the open-source software PyMOL.

### Construction of the Δ*iscR* strain

The in-frame deletion of *iscR* gene from the *C. crescentus* NA1000 chromosome was obtained by allelic replacement. The flanking regions of the *iscR* gene were amplified by PCR with primers 01942IF1/01942IF2 and 01942IF3/01942IF4 (all primers mentioned henceforth are described in Supplementary Table S3). These two fragments were then cloned *in tandem* into the suicide vector pNPTS138 (M.R.K. Alley, unpublished) using the In-Fusion cloning kit (TaKaRa Bio) and transformed into competent *E. coli* DH10B cells. The vector was introduced into *E. coli* S17-1 and subsequently into *C. crescentus* NA1000 via conjugation. The kanamycin-resistant colonies obtained were grown in PYE for 48 h, followed by plating on PYE 3% sucrose. Kan-sensitive clones were investigated by PCR with primers 42CompF/42CompR, and a clone presenting a single 765-bp amplified fragment was selected as an *iscR* deletion mutant for further characterization.

### Construction of *lacZ* transcriptional fusions and β-galactosidase activity assay

Different fragments of the *iscR* promoter region were amplified by PCR, using the genomic DNA of *C. crescentus* NA1000 as a template and specific forward primers pLacZFA, pLacZFB, and pLacZFC combined with the reverse primer pLacZ2R. The amplified fragments were cloned into the pRK*lacZ*290 vector (Gober and Shapiro, 1992), generating vectors p*lacZ*FragA, p*lacZ*FragB, and p*lacZ*FragC. The plasmids were subsequently transferred into *C. crescentus* strains NA1000, Δ*iscR*, Δ*fuR*, and Δ*oxyR* by conjugation with *E. coli* S17-1. To evaluate the activity of the promoter regions under study, the activity of the β-galactosidase enzyme was measured, using the method described by (Miller, 1972).

Strains containing these reporter plasmids were grown in PYE/Tet and expression was analyzed at logarithmic phase (OD_600 nm_ = 0.5) under different experimental conditions: in the presence of 100 μM DP for 2 h, or 60 μM H_2_O_2_ for 30 min.

### Streptonigrin assay

The streptonigrin (SNG) sensitivity tests were carried out as described in Nachin et al. (2001) and Justino et al. (2007). The wt and Δ*iscR* strains were grown at 30°C in PYE, diluted to OD_600nm_ = 0.1, and aliquoted into new tubes, which received 0.05 μg/mL, 1 μg/mL, or no streptonigrin (Sigma-Aldrich), respectively. The cultures were incubated for 24 h at 30°C, serially diluted, plated on PYE medium and incubated for 48 h at 30°C to determine CFU/ml.

### Reverse transcription quantitative real-time PCR (RT-qPCR)

For RT-qPCR assays, cultures were grown in PYE medium up to exponential phase (OD_600 nm_ = 0.5), 1 mL aliquots were treated with TRIzol^®^ (Invitrogen Life Technologies), and the RNA was extracted following the manufacturer’s instructions. RNA (2 μg) was treated with one unit of DNAse I (Invitrogen) and a negative control PCR was carried out without reverse transcriptase using primers for the *rho* gene (Supplementary Table S3). cDNA synthesis was performed using the SuperScript III First-Strand Synthesis Kit for RT-qPCR (Invitrogen). RT-qPCR experiments took place using Power SYBR Green and PCR master Mix (Applied Biosystems), using the CCNA_01991 or *rho* genes as internal controls for reference. RNA was amplified in duplicate for each biological replicate with primers designed for each gene, and reactions were performed in the StepOnePlus real-time PCR system (Thermo Fisher Scientific). The relative differential expression was calculated using the 2^−ΔΔCt^ relative expression quantification method (Livak and Schmittgen, 2001).

### Total RNA extraction and sequencing (RNA-seq)

Three independent biological replicates from strains *C. crescentus* NA1000 (*n* = 3) and Δ*iscR* (*n* = 3) were grown in PYE medium up to OD_600 nm_ = 0.5 at 30°C with agitation at 200 rpm. Total RNA was extracted from 10 mL-cultures essentially as previously described (de Araújo et al., 2021). rRNA depletion and cDNA libraries were prepared using the Illumina Stranded Total RNA Ligation Kit with RiboZero Plus (Illumina) and subsequently sequenced using the NextSeq 500/550 Mid Output Kit v2.0 (Illumina) on the Illumina NextSeq 500 (Illumina). RNA sequencing was done in the Core Facility for Scientific Research – University of São Paulo (CEFAP-USP/GENIAL).

### Construction of the IscR-3xFLAG strain

A DNA fragment containing the coding region of the *iscR* gene along with 1 kb upstream and downstream flanking regions was obtained by PCR using the Q5 DNA polymerase (New England Biolabs), genomic DNA from strain NA1000, and primers IscR Fw/IscR Rv. This blunt fragment was cloned using Zero Blunt™ PCR Cloning Kit (Thermo) and the recombinant pCR™-Blunt plasmid was used as template in a PCR reaction with outward primers that hybridized in the 3’end of the *iscR* coding region, containing 1.5xFLAG coding sequence in each of their 5′ regions. After ligating with T4 DNA ligase (Thermo), this reaction yielded a pCR™-Blunt plasmid containing a 3x FLAG coding sequence inserted in frame with the C-terminal region of IscR. The correct fusion was confirmed by DNA sequencing using the BigDye™ Terminator v3.1 Cycle Sequencing Kit (Thermo). The 1.7 kb-DNA fragment containing the *iscR*-*3xFLAG* gene was obtained by digestion with EcoRI (Thermo), cloned into the suicide vector pNPTS138 and inserted in *E. coli* S17-1 by transformation. The plasmid was transferred to *C. crescentus* NA1000 by conjugation, and after double recombination, the *iscR* gene was substituted by the *iscR*-*3xFLAG* version.

### Chromatin immunoprecipitation assay (ChIP-seq)

The protocol was followed essentially as described by (Gebhardt et al., 2020; Gozzi et al., 2022) in a threefold larger scale. Briefly, cultures from the IscR-3xFLAG strain were freshly diluted to OD_600 nm_ = 0.1 in 180 mL PYE at 30°C at 200 rpm. After reaching an OD_600 nm_ = 0.3, cultures continued to grow in the normal condition (PYE medium, *n* = 3) or under iron deficiency condition (PYE with 100 μM DP for 2 h, *n* = 3). Crosslinking was performed with formaldehyde to a final concentration of 1%, the cells were further incubated for 30 min, and glycine was added to 0.125 M for 15 min at room temperature. Cells were disrupted by sonication long enough to obtain DNA fragments between 250 and 500 nt, followed by centrifugation for 20 min at 12,000 × *g* at 4°C. 3xFLAG-tagged IscR was immunoprecipitated using 200 μL of Anti-FLAG M2 affinity gel beads (Sigma-Aldrich) at 4°C overnight. After washing the resin 5× at 4°C, the protein was eluted from the resin in 150 μL of elution buffer.

After obtaining the DNA-ChIP fraction, purification was carried out using the Qiaquick PCR purification kit (Qiagen) following the manufacturer’s instructions and eluted using 50 μL of MilliQ H_2_O. DNA concentration and fragment size was determined using BioDrop (Biochrom) and Bioanalyzer (Agilent). The purified DNA was used to construct libraries with the NEBNext^®^ Ultra™ II DNA Library Prep Kit for Illumina^®^, and the libraries were sequenced using the NextSeq 500/550 Mid Output Kit v2. 0 (150 cycles) (Illumina) on an Illumina NextSeq 500 (Illumina).

### Expression and purification of IscR-His and electrophoretic mobility shift assay (EMSA)

The *iscR* gene was amplified by PCR with primers pETIscR1/pETIscR2, cloned into vector pET28a in frame with the 6-histidine tag codons at the carboxi terminus, and transformed into *E. coli* BL21(DE3). The IscR-His protein expression was induced with 300 μM IPTG for 2 h and the bacterial cell pellet obtained from a 1-liter LB culture was resuspended in 40 mL of buffer A (50 mM HEPES, 100 mM NaCl, pH 8). Subsequent sonication was performed on ice at 37% amplitude for 4 min (30 s ON/30 s OFF) followed by clarification via centrifugation at 25,000 × *g* for 15 min. The clarified extract was loaded onto a pre-equilibrated 5 mL HiTrap Heparin HP column, utilizing an AKTAPrime Plus FPLC system with a flow rate set at 4 mL.min^−1^. The column was washed with buffer A until the UV signal (measured at 280 nm) reached baseline and a linear gradient was applied, ranging from 0 to 100% of buffer B (50 mM HEPES, 1 M NaCl, pH 7), over a course of 10 column volumes (CV). Fractions collected during this gradient elution were subsequently analyzed via SDS-PAGE.

Reddish fractions containing IscR-His were combined, diluted 10-fold in buffer C (50 mM HEPES, 100 mM NaCl, pH 7), and loaded onto a pre-equilibrated 2 mL HiTrap SP FF column, maintaining a flow rate of 2 mL.min^−1^. The column was washed with 10 CV and subjected to a linear gradient elution, starting from 0% and ending at 100% of buffer B. Fractions were likewise analyzed via SDS-PAGE and those containing IscR-His were then diluted twice with distilled water, quantified through UV absorption at 280 nm (considering ε_IscR_ = 11,585 M^−1^ cm^−1^ and _MWIscR-His_ = 21.04 kDa) and stored at 4°C for subsequent use. The protein sample was also loaded into quartz cuvettes and subjected to UV–Vis absorption scanning from 300 to 900 nm (integration time = 0.01 s, interval = 1 nm) with a BioChrom BioDrop Duo spectrophotometer. As a control for the apo state, the protein sample was incubated with EDTA and DP at 100X and 10X the protein concentration, respectively, for 5 min at room temperature.

The electrophoretic mobility shift assay was performed using a 442 bp probe from the *iscR* promoter region that was obtained by amplification from *C. crescentus* NA1000 DNA with the primers p*lacZ*FA and p*lacZ*2R. The probes for the other promoters were obtained with specific primer pairs generating DNA fragments of approximately 250 bp. DNA binding assay was performed in 20 μL in 10 mM HEPES, 40 mM KCl, 1 mM MgCl_2_, 1 mM MnCl_2_, 5% glycerol, salmon sperm DNA (0.1 mg.ml^−1^), 500 ng of the DNA probe and increasing amounts of purified IscR-His protein (5, 250, and 500 nm). After incubation for 30 min at room temperature, the samples were analyzed by electrophoresis in a 5% polyacrylamide gel in 1X Tris-borate buffer (TBE) for 1 h at 20 mA. The detection of bands was carried out using the Electrophoretic Mobility Shift Assay kit (Thermo Fisher) or ethidium bromide.

### Sequence data analyses

RNA and DNA sequencing data were processed using the frtc pipeline^1^ (Ten-Caten et al., 2018). We aligned the reads to *C. crescentus* NA1000 genome using the assembly ASM2200v1.

We used uniquely aligned RNA-Seq reads for gene differential expression analysis as in (de Araújo et al., 2021), using DeSeq2 (Love et al., 2014) and custom R scripts. We analyzed COG category enrichment as in (de Araújo et al., 2021). We removed the genes belonging to the genomic island that is absent only in parental strain (CCNA_00460, CCNA_00464 to CCNA_00482, CCNA_03921, CCNA_03922 and CCNA_03998) from the list of differentially expressed genes (see section “Construction of the *ΔiscR* strain”).

We used uniquely aligned ChIP-Seq reads as input for peak-calling with MACS2 (v2.2.6) (Zhang et al., 2008). Peak-calling was done individually for each replicate, using the parameters -g 4,047,433 [effective genome size, estimated with the khmer program (v2.1.1)] (Döring et al., 2008; Brown et al., 2015; Irber and Brown, 2016) and-f BAMPE, as the data is from paired-end sequencing. We then used the DiffBind R package (v3.12.0) (Stark and Brown, 2011; Ross-Innes et al., 2012) to (i) find significant MACS2-called peaks among the replicates and (ii) search for differentially bound regions by IscR between treatments. Reads in significant peaks were counted with dba.count command with parameters: bSubControl = F, minOverlap = 2, filter = 1,000, filterFun = mean, summits = 50. We performed the differential binding analysis using the treatment (addition of DP to PYE medium) as the contrast and the DBA_DESEQ2 method for the calculations.

We used the Integrative Genomics Viewer (IGV) (Robinson et al., 2011) to visualize and integrate all data. Comparison of the IscR regulon with previously published Fur regulon used data from da Silva Neto et al. (2013) and Leaden et al. (2018).

### Statistical analysis

All experiments were conducted using a minimum of two independent biological replicates for each experimental condition, and in the qRT-PCR experiment two technical replicates were also employed. Mean and standard deviation were calculated from the data obtained from biological replicates, and to determine the statistical significance between different experimental conditions, the Student’s *t*-test (unpaired *t*-test) was used, with a significance level set at *p* < 0.05.

## Results

### The *isc-suf* operon organization is conserved in Alphaproteobacteria

**Figure 1:**
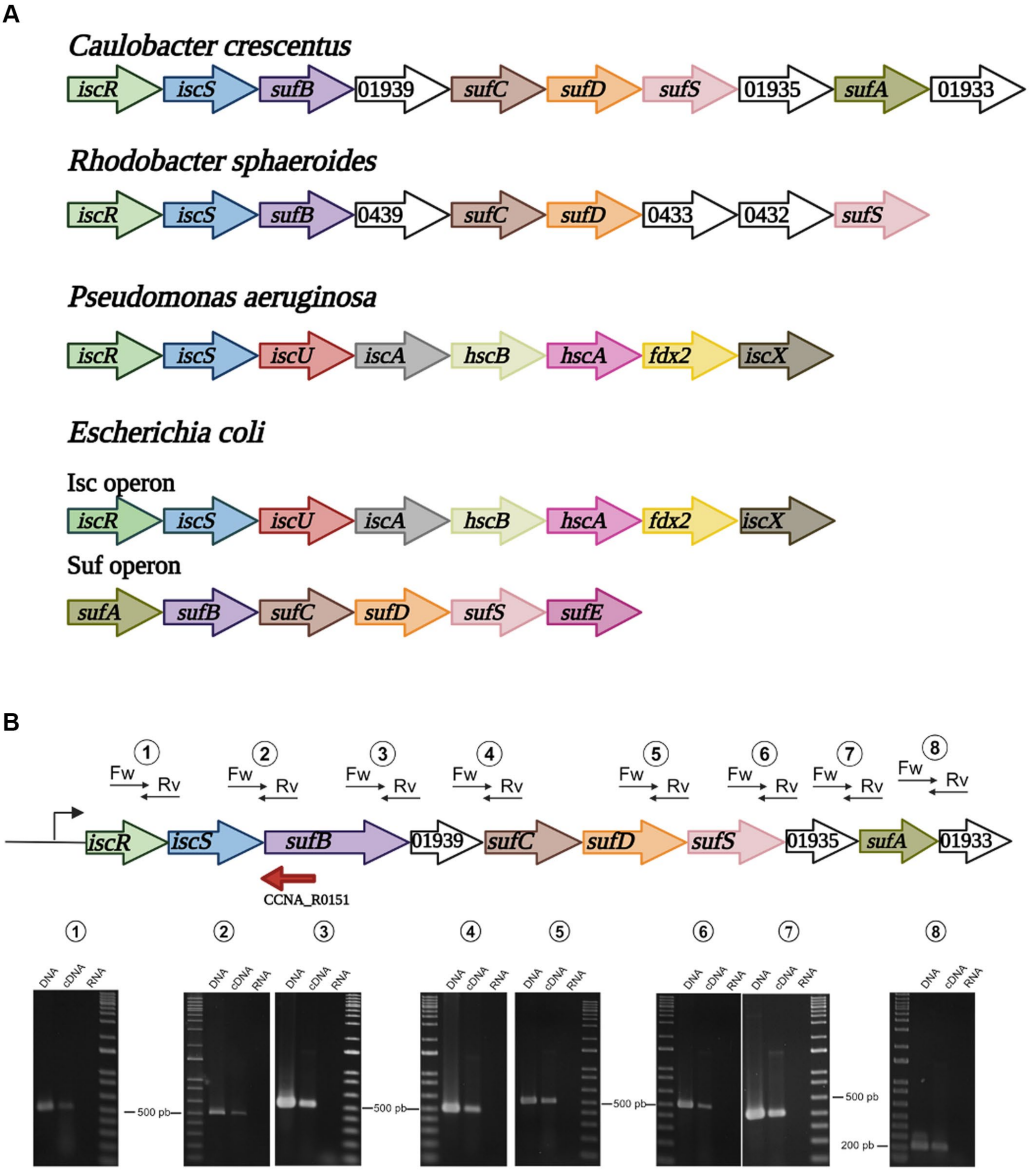
Organization of the *isc* and *suf* operons in selected bacteria. **(A)** Alphaproteobacteria such as *C. crescentus* and *R. sphaeroides* possess a single operon containing *iscRS* followed by *suf* genes. In Gammaproteobacteria, *P. aeruginosa* has a single *isc* operon, while *E. coli* and Enterobacteria have two distinct operons, *isc* and *suf*. **(B)** Schematic drawing showing each gene, and the positions of the primer pairs designed to amplify some of the genes junctions. RT-PCR using each primer pair with *C. crescentus* NA1000 total genomic DNA (DNA), cDNA from total RNA (cDNA) or total RNA (RNA). Marker, 1 kb Plus DNA marker. The sizes of the nearest bands are indicated for reference. Created in BioRender. Santos, N. (2024) BioRender.com/o98n394.

*Caulobacter crescentus* and other Alphaproteobacteria have a single operon encoding putative [Fe-S] cluster biosynthesis proteins that contains *iscRS* followed by orthologous of *E. coli suf* genes (Figure 1A and Supplementary Figure S1). IscS and SufS consist of cysteine desulfurases responsible for removing the sulfur groups from L-cysteine generating L-alanine and sulfur. The latter is then transferred to a conserved cysteine residue before subsequent transfer to scaffold proteins (Blanc et al., 2015). When comparing *C. crescentus* and *E. coli* genomes by Reciprocal Best Hits analysis, we were able to identify the *C. crescentus* genes *CCNA_01941* and *CCNA_01936* as putative orthologs for *E. coli iscS* (34.9% identity, YP_026169.1) and *sufS* (49.8% identity, NP_416195.1), respectively. To obtain a more robust hypothesis of orthology, we also modeled the tertiary structure of the proteins CCNA_01941 and CCNA_01936 with AlphaFold2. According to Fujishiro et al. (2022), general protein architecture is observable among members of the cysteine desulfurase family, but local structural features can distinguish them. CCNA_01936 presented a shorter catalytic loop and a typical β-hairpin region in the vicinity of the dimer interface. Both features are only observed in SufS-like proteins while longer catalytic loops and the absence of the β-hairpin are characteristic of IscS-like ones, as seen in CCNA_01941 (Supplementary Figure S2). Also, when analyzing the synteny of the *iscS* gene among several genomes from the Pseudomonadota phylum, we were able to determine that other Alphaproteobacteria present the same gene organization in this operon (Supplementary Figure S1). Moreover, in Betaproteobacteria and Gammaproteobacteria other than the Enterobacteriaceae, *iscRS* is part of the *isc* operon, like in *E. coli* (Figure 1A and Supplementary Figure S1).

The other genes downstream of *iscS* in *C. crescentus* are orthologs of the *E. coli suf* genes, so herein we will refer to this operon as the *isc-suf* operon for simplicity. The *C. crescentus sufB*, *sufC*, *sufD* and *sufS* orthologs are in the same order of the *E. coli suf* operon, except for a small ORF between *sufB* and *sufC* annotated as a putative ADP-ribosylglycohydrolase (Figure 1A). Downstream of *sufS*, CCNA_01935 is annotated as a FeS assembly SUF system protein (KEGG), followed by two ORFs. CCNA_01934 encodes a putative ortholog of *sufA* (50% similarity) and CCNA_01633 encodes a conserved hypothetical 66 aa-protein containing five cysteines. To verify that the genes were in fact co-transcribed, we designed primer pairs to amplify some of the gene junctions by RT-PCR. All the primer pairs generated an amplified band, confirming that all genes are part of the same operon (Figure 1B).

### IscR regulates the expression of the *C. crescentus isc-suf* operon in response to iron

**Figure 2:**
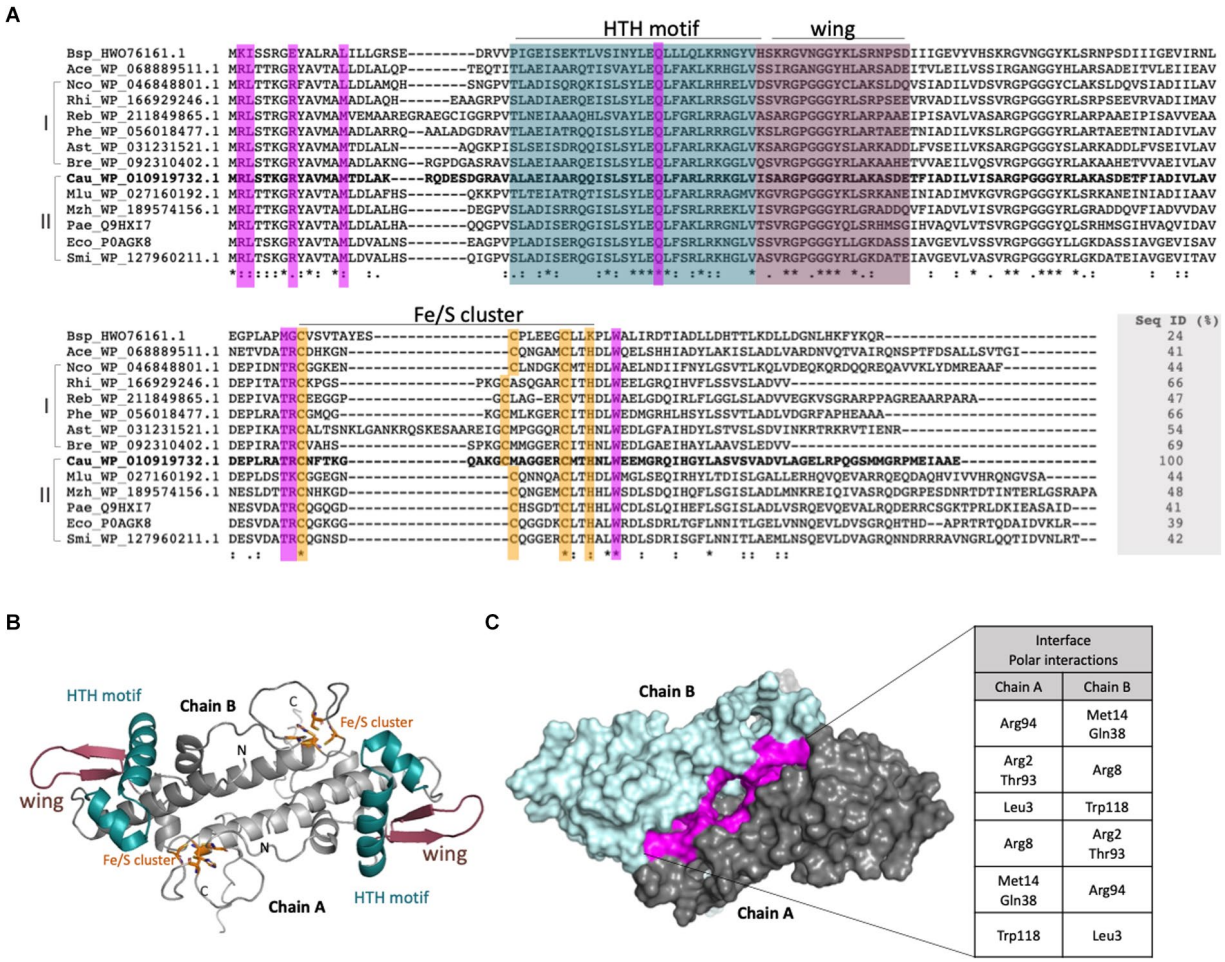
Sequential and structural characterization of the *C. crescentus* IscR. **(A)**. Structural alignment of putative IscR proteins from selected bacteria was made with Expresso from webserver T-Coffee (Notredame et al., 2000). Bsp_HWO_76161.1: *Bacillus* sp.; Ace_WP_068889511.1: *Acinetobacter celticus*; Nco_WP_046848801.1: *Nitrosomonas communis; Rhi_*WP_166929246.1: *Rhizomicrobium electricum*; Reb_WP_211849865.1: *Roseomonas eburnea*; Phe_WP_056018477.1: *Phenylobacterium* sp.; Ast_WP_031231521.1: *Asticcacaulis* sp. YBE204; Bre_WP_092310402.1 *Brevundimonas viscosa*; Cau_YP_002517315 *Caulobacter crescentus* NA1000; Mlu_WP_027160192.1 *Methylobacter luteus*; Mzh_WP_189574156.1 *Marinobacter zhanjiangensis*; Pae_NC_002516.2: *Pseudomonas aeruginosa*; Eco_NP_417026.1 *Escherichia coli*; Smi_WP_127960211.1: *Serratia microhaemolytica*. I and II: Sequences were classified in two groups according to the phylogenetic tree. Residues belonging to the HTH motif and wing are colored in deep cyan and dark purple, respectively. Residues involved in the Fe-S cluster binding (3 Cys and 1 His) are shown in orange and residues that form an interface between both monomers are shown in purple. **(B)** Model of the IscR generated with AlphaFold2. The dimer is shown in cartoon representation with the motifs and residues colored according to **(A)**. **(C)** Dimer of IscR in surface mode highlighting the interface between the monomers in purple. The polar interactions performed are described in the table.

The protein encoded by CCNA_01942 possesses all the conserved structural features of IscR orthologs, which include the winged HTH motif for DNA recognition and the four residues that form the 2Fe-2S cluster binding (Cys^95^, Cys^105^, Cys^112^, and His^115^) (Figure 2A). The three-dimensional model of IscR produced in AlphaFold2 shows a dimer with rotational symmetry in which the two Fe/S clusters are arranged on opposite sides, as well as the HTH and wing motifs (Figure 2B). At least 8 residues of each monomer are involved in the maintenance of the dimeric interface through polar interactions (Figure 2C).

**Figure 3:**
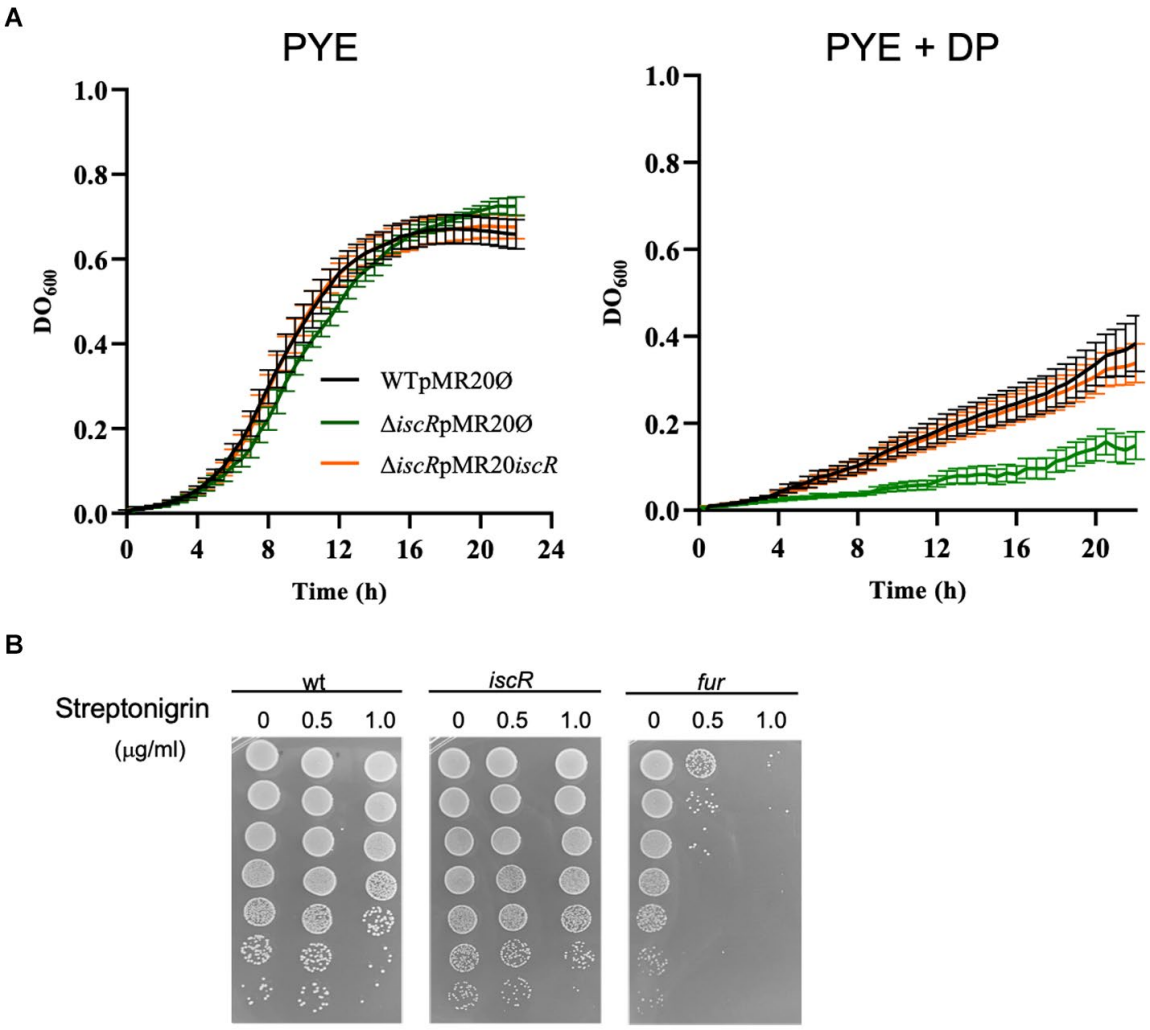
Phenotypic analysis of the *iscR* mutant. **(A)** Growth of strains NA1000 (wt) containing the empty pMR20 plasmid (black line), Δ*iscR* containing the empty pMR20 plasmid (dark green line) and Δ*iscR* pMR20-*iscR* (blue line) was carried out in PYE and in PYE with the addition of 100 μM DP at 30°C with agitation. Growth was assessed by measuring the OD_600 nm_ of six independent biological replicas. **(B)** The cultures of strains NA1000 (negative control), Δ*iscR* and Δ*fur* (positive control) were grown in PYE up to midlog phase and incubated with 0 (control), 0.5 μg/mL or 1 μg/mL Streptonigrin for 24 h. The cultures were subjected to serial dilutions followed by plating on PYE and after 3 days at 30°C CFU counts were taken. These images are representative of 2 independent biological replicates.

To characterize the role of IscR, we generated a strain with an in-frame deletion of the *iscR* gene (Δ*iscR*). The mutant growth in PYE medium was slightly slower than the wt, but severely impaired in iron depletion (addition of DP) (Figure 3A). The expression of the *iscR* gene *in trans* from the low-copy number plasmid pMR20 directed by its own promoter restored the wt phenotype (Figure 3A). To verify whether the mutant had an imbalance in intracellular iron concentration, we performed a streptonigrin sensitivity test (Figure 3B). Streptonigrin is an antibiotic that requires a redox-active metal to be fully active and has been used as an indirect measurement of intracellular iron concentration (Zik et al., 2022). As seen in Figure 3B, the Δ*iscR* mutant does not show an increased sensitivity to 0.5 or 1.0 μg/mL streptonigrin when compared to wt, differently from the high sensitivity of the *fur* mutant (positive control). These results indicate that the lack of *iscR* does not generate an accumulation of intracellular iron, although cells present a much slower growth rate at low iron levels, probably due to incorrect gene expression for iron starvation response. We have also evaluated the response of the *iscR* mutant to oxidative stress generated by either H_2_O_2_ or paraquat (Supplementary Figure S3). Although the mutant seems to be slightly more sensitive to paraquat than the wt after 15 min, at all the other time points the phenotype was the same. These results indicate that the *iscR* mutant is not more sensitive to oxidative stress than the wt strain.

**Figure 4:**
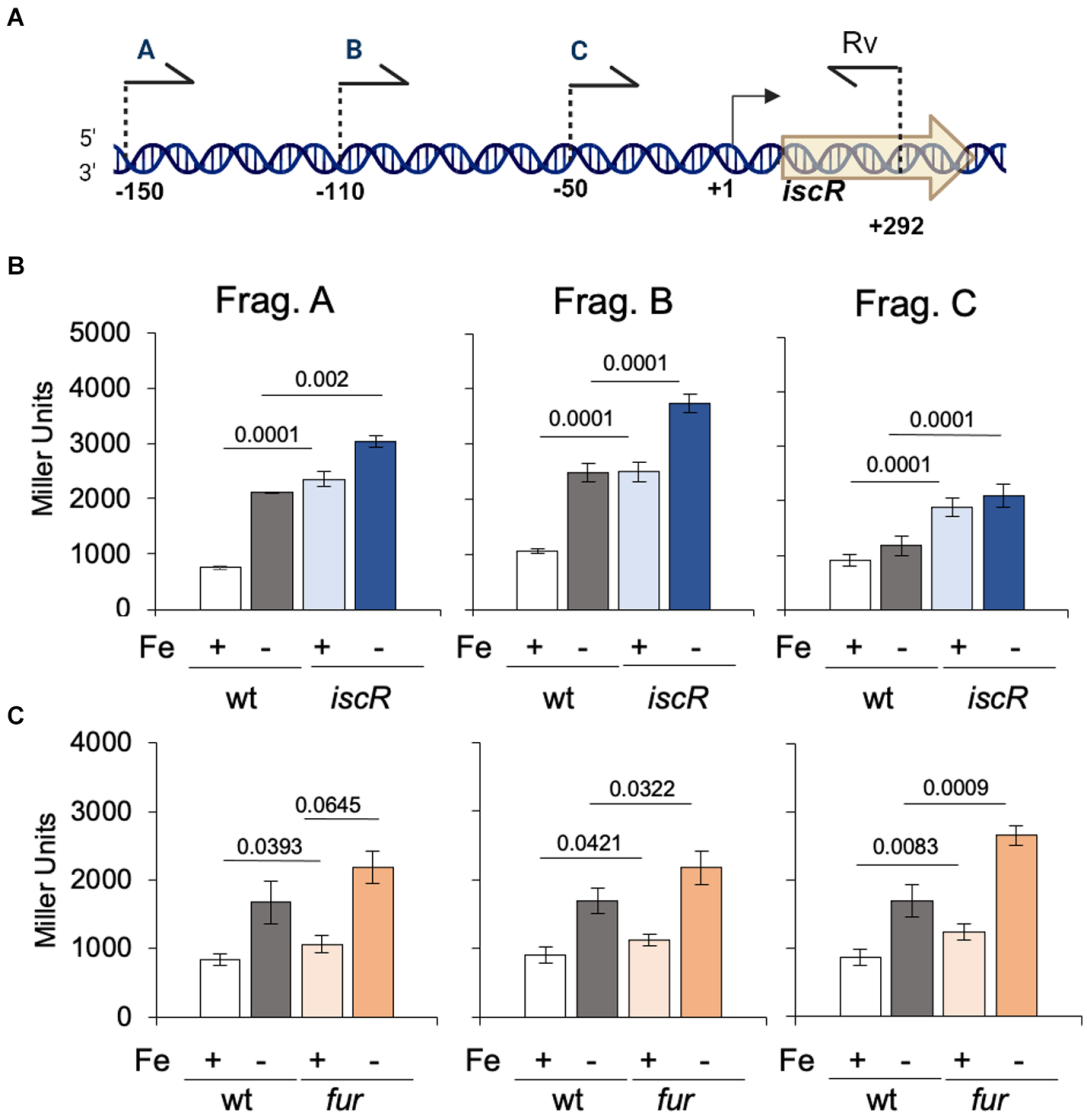
Regulation of the *isc* transcription by IscR. **(A)** Scheme of the 5′ limits of the *isc* regulatory region used to generate fragments A, B, and C cloned in front of the *lacZ* gene in vector pRKlacZ290. Numbers indicate the positions relative to the transcriptional start site (+1). Created in BioRender. Santos, N. (2024) BioRender.com/n65i911. **(B)** Cultures of NA1000 and the Δ*iscR* strains containing the plasmid with each distinct fusion of the *isc* promoter to the *lacZ* gene were grown in PYE medium up to midlog phase. The cultures were divided in two tubes, containing (Fe−) or not (Fe+) 100 μM 2′-2 dipyridyl and further incubated for 2 h (*n* = 6). **(C)** Cultures of NA1000 and Δ*fur* strains containing the three transcriptional fusions were grown in PYE medium up to midlog phase. The cultures were divided in two tubes, containing PYE with no addition (Fe+), or added of 100 μM 2′-2 dipyridyl (Fe−) and further incubated for 2 h (*n* = 4). Promoter activity was determined by beta-galactosidase activity assays and expression is shown in Miller units (36). Statistical analysis of significance was calculated using Student’s *t-*test (*p-*values are shown above each bar).

IscR has been reported to regulate the *isc* operon in several bacteria (Schwartz et al., 2001; Nesbit et al., 2009; Remes et al., 2015) and in *C. crescentus* the *isc-suf* operon was upregulated in low iron condition (da Silva Neto et al., 2013). In *C. crescentus iscR* is the first gene of the *isc-suf* operon, so we measured the expression driven by the *isc* promoter in the Δ*iscR* strain in conditions of sufficient or scarce iron (Figure 4). For that, three transcriptional fusions to the *lacZ* gene were constructed, containing successive deletions of the *isc* regulatory region (Figure 4A), as a tentative to map the sequences involved in gene regulation. The results showed that expression driven by the larger fragment containing 150 bp upstream of the *iscR* transcription start site (TSS) (Figure 4B, Fragment A) increases about 3-fold in low iron while its basal levels are already high to the same level in the Δ*iscR* mutant. Moreover, gene expression was further increased when the Δ*iscR* strain was in low iron conditions. These results indicate that IscR is repressing its own transcription in high iron (as a holo-IscR protein) but there is a second mechanism of regulation occurring at a low-iron condition. This increase can still be observed in construction B, which starts at position-110 bp, but is not seen with construction C that contains only the promoter (starting at position-49) (Figure 4B). These results suggest that an activator might bind upstream of the-35 region. We then tested whether the *isc-suf* operon upregulation under iron starvation was affected in the *fur* mutant (Figure 4C). The results showed that transcription driven from all fragments was increased in iron depletion, and that in the *fur* mutant the levels of expression were similar to wt. These data corroborate previous results (da Silva Neto et al., 2013) showing that the *isc-suf* operon was not directly regulated by Fur.

**Figure 5:**
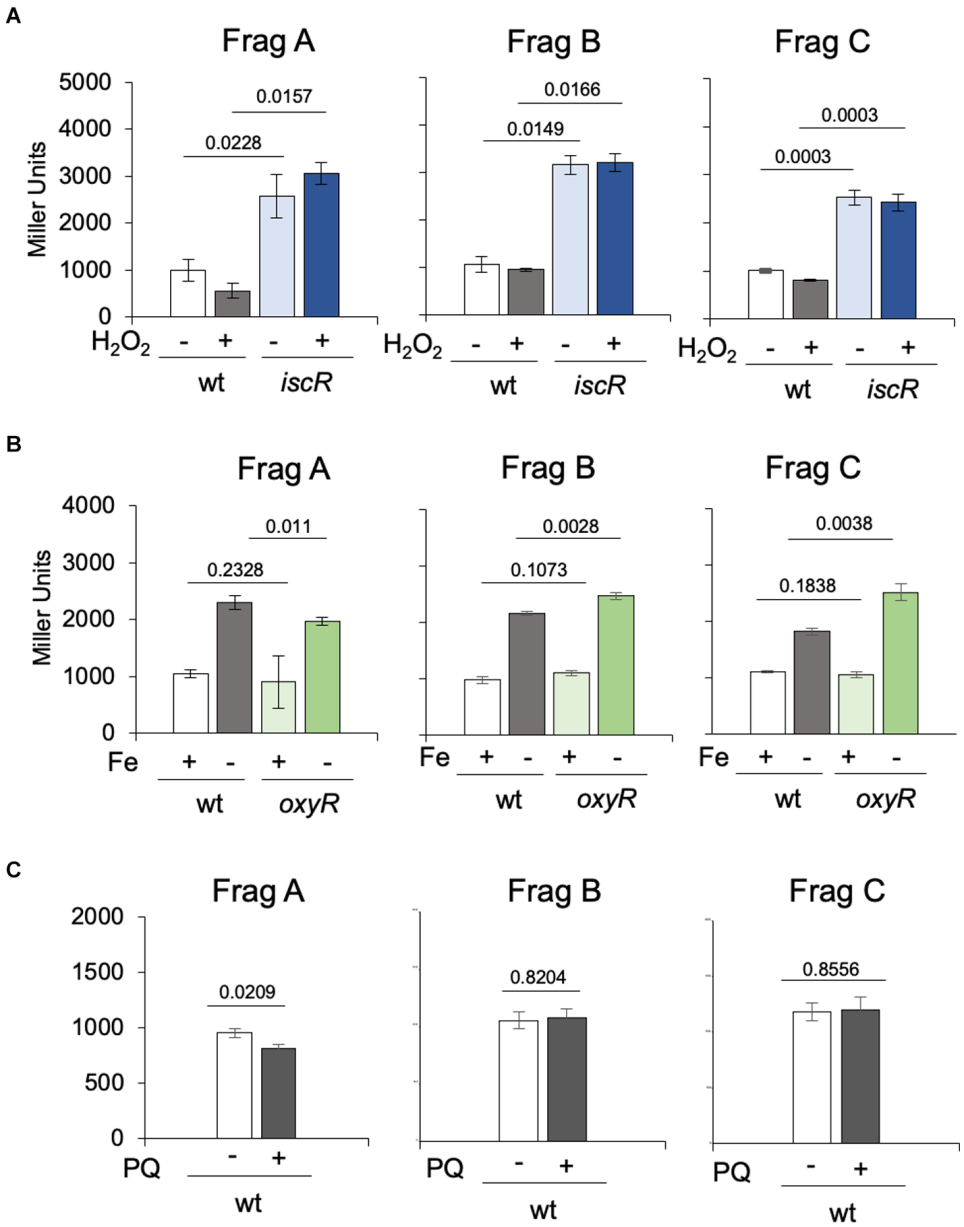
Regulation of the *isc* transcription in response to oxidative stress and in the *oxyR* mutant. **(A)** Cultures of NA1000 and Δ*iscR* strains in PYE medium containing the three transcriptional fusions from Figure 3A in PYE medium were incubated (+) or not (−) with 80 μM H_2_O_2_ for 30 min (*n* = 2). **(B)** Cultures of NA1000 and Δ *oxyR* strains with the transcriptional fusions were grown in PYE medium up to midlog phase. The cultures were divided in two tubes, containing PYE with no addition (Fe+), or added of 100 μM 2’-2 dipyridil (DPP) (Fe-) and further incubated for 2h (*n* = 4). **(C)** Each transcriptional fusion was incubated with 50 μM paraquat (PQ) for 2 h (*n* = 3). Promoter activity was determined by beta-galactosidase activity assays and expression is shown in Miller units (36). Statistical analysis of significance was calculated using Student’s *t-*test (*p*-values are shown above each bar).

To evaluate the transcription levels when cells are exposed to oxidative stress, the wt and Δ*iscR* strains were incubated with 80 μM H_2_O_2_ for 30 min. The results showed that H_2_O_2_ has no effect on *iscR* expression, and transcription is derepressed in the Δ*iscR* mutant, as expected (Figure 5A). These results are in accordance with the previously reported *C. crescentus* oxidative stress stimulon, where *iscR* was not upregulated after 5 min or 15 min after H_2_O_2_ addition (Silva et al., 2019). To ascertain whether the oxidative stress regulator OxyR contributed to the *isc* induction, the same analysis was carried out using the *oxyR* mutant in different iron concentrations with the three constructs (Figure 5B). The results showed that expression in iron-depleted conditions was higher than in iron sufficiency in all constructs irrespective of the strain (Figure 5B), and the absence of OxyR had no effect on *isc* transcription. We have also evaluated if the gene responded to superoxide, incubating the cultures with 50 μM paraquat for 2 h prior to the assay, and no induction was observed (Figure 5C). Taken together, these results show that the *isc-suf* operon is not regulated in response to oxidative stress.

### Identification of the IscR regulon

**Figure 6:**
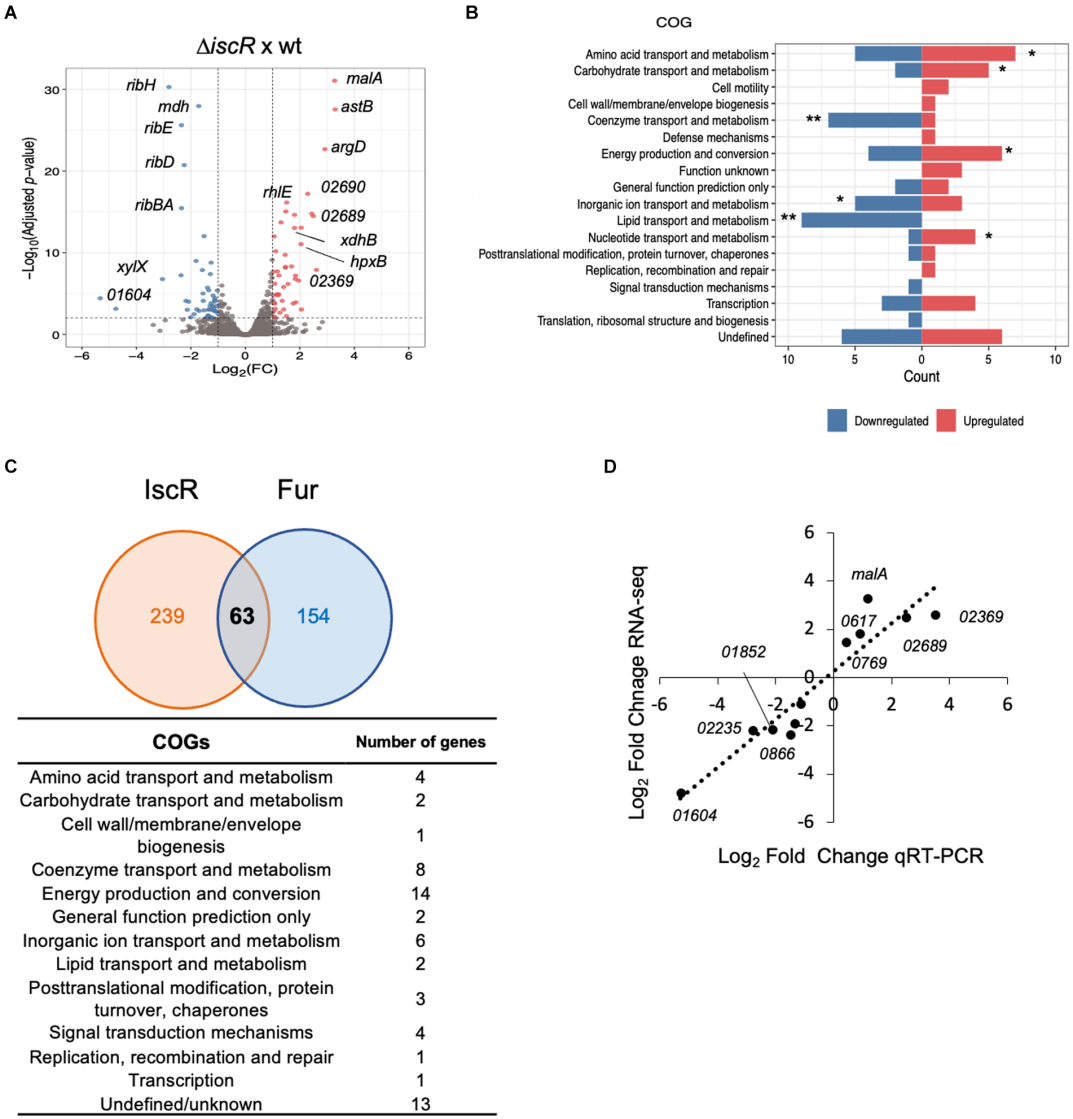
Global transcriptomic analysis of the IscR regulon. **(A)** Volcano plot with the distribution of DEGs in the Δ*iscR* versus wt. Colored dots indicate genes that were differentially expressed in the mutant: blue, downregulated; red, upregulated. **(B)** Functional characterization of DEGs obtained according to the Cluster of Orthologous Genes (COG). The categories defined to be overrepresented in each analysis are those with the Benjamini-Hochberg adjusted *p*-value <0.05, as indicated: * adjusted *p*-value <0.05; ** adjusted *p*-value <0.01. **(C)** Intersection between the predicted *C. crescentus* IscR and Fur regulons. The number of genes comprising the IscR regulon was calculated as a sum of the DEGs identified in the RNA-seq experiment and genes with an upstream peak of IscR binding, identified in the ChIP-seq experiment. The Fur regulon was obtained from (6, 26, 27). All the genes in the operons were considered in this comparison. The categories of the genes predicted to belong to both regulons were obtained according to COG. **(D)** The relative amount of mRNA for several DEGs in the wt and in the Δ*iscR* mutant was determined by RT-qPCR using primer pairs that amplify each ORF. A correlation analysis between the differential expression obtained by RNA-seq and RT-qPCR is shown (2 biological samples and 2 technical samples).

**Figure 7:**
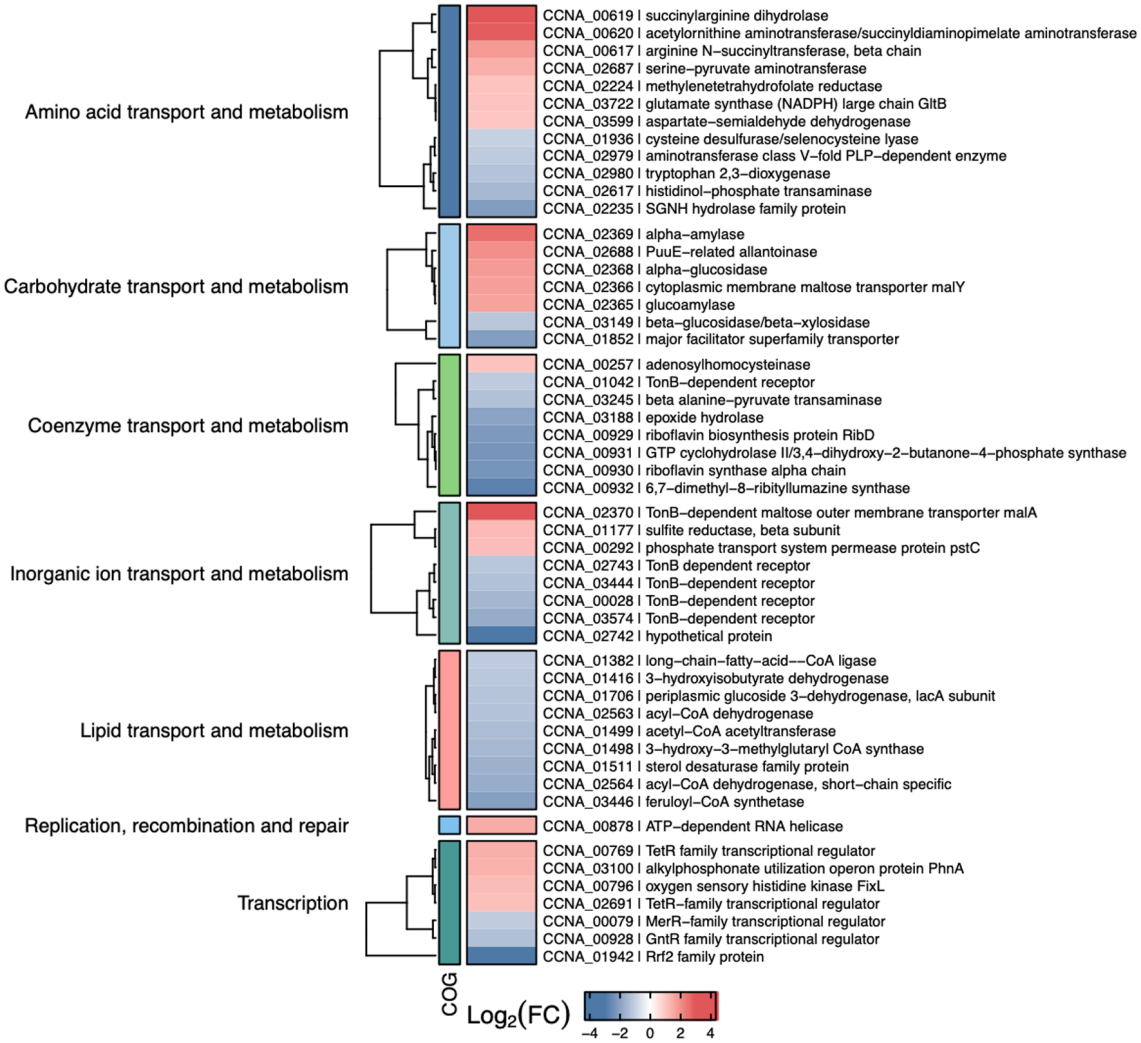
Selected differentially expressed genes in the *iscR* mutant. Heat map showing some of the DEGs determined by RNA-seq analyses of Δ*iscR* x wt. The colors indicate the log_2_[Fold Change] according to the legend at the bottom and the description of the categories on the left. “Figure made using R package ComplexHeatmap (ref – https://academic.oup.com/bioinformatics/article-abstract/32/18/2847/1743594).

To determine the importance of IscR as a transcriptional regulator in *C. crescentus*, we carried out a global transcriptional profiling of the Δ*iscR* mutant vs. wt, grown in PYE at 30°C with agitation, in a condition of sufficient iron. The transcriptomic analysis identified 94 DEGs, with 47 upregulated (log_2_FoldChange ≥ 1.0 and adjusted *p*-value <0.01) and 47 downregulated genes (log_2_FoldChange ≤ −1.0 and adjusted *p*-value <0.01) in the Δ*iscR* mutant (Supplementary Table S4 and Figure 6A). The DEGs were categorized according to the Cluster of Orthologous Genes (COG) (Figures 6B, 7). The categories Coenzyme Transport and Metabolism, Inorganic Ion Transport and Metabolism and Lipid Transport and Metabolism were overrepresented among the downregulated genes, while Amino acid transport and metabolism, Carbohydrate transport and metabolism, Energy Production and Conversion and Nucleotide Transport and Metabolism were more predominant among the upregulated genes. The expression of some of the DEGs was determined by RT-qPCR and the results validated the RNA-seq expression profile (Figure 6D).

Among the upregulated genes there were operons encoding enzymes for arginine biosynthesis (CCNA_00617–00620), maltose transport (CCNA_02365–02370), and two operons for purine metabolism (CCNA_02687–02690 and CCNA_02699–02701) (Supplementary Table S4). Moreover, operons encoding cytochromes *cb* and *bd* from the electron transport chain (CCNA_01468–01471 and CCNA_00801–00802) were also upregulated. While some of these genes encode [Fe-S]- or iron-binding proteins, like CCNA_02690, FixG, CysI, CcoO, CcoP and GltB, others encode proteins binding to different divalent cations such as zinc and molybdenum. Among the most downregulated genes it is of note the operon encoding enzymes for the synthesis of riboflavin (CCNA00929-00932) and the *qoxA*-*D* operon (CCNA_01851–01849) that encodes a Heme/copper-type cytochrome/quinol oxidase (Supplementary Table S4). Accordingly, several genes encoding flavin adenine dinucleotide binding enzymes were also downregulated. Several genes involved in lipid transport and metabolism were downregulated, encoding distinct dehydrogenases and NAD-or FAD-binding enzymes (Figure 7).

A very intriguing result was that almost none of the genes belonging to the *isc-suf* operon were differentially expressed in the Δ*iscR* mutant, although we observed a threefold increase in expression driven by the *isc-suf* promoter in the Δ*iscR* mutant (Figure 4B). The RNA-seq results showed a twofold increase for *iscS*, but the difference was not considered statistically different by our parameters (adjusted *p*-value <0.01), and the *sufS* gene was downregulated in the *iscR* mutant. To clarify this, we investigated the variation in mRNA levels for several genes of the *isc-suf* operon by RT-qPCR (Supplementary Figure S5A). We observed that except for *iscS*, that is highly upregulated in the *iscR* mutant, the levels of the *suf* genes do not change between the strains. This result indicates that there must be a second layer of regulation that occurs after *iscS*, at the mRNA level.

To try to map a little more precisely where this regulation is occurring, we have designed 4 primer pairs for the beginning, middle and end of the *iscS* gene (Supplementary Figure S5B), and we determined the amount of transcript in each region by RT-qPCR in the Δ*iscR* mutant and wt. The results showed that the relative amount of mRNA from the beginning of the *iscS* gene is around 10-fold. However, this relation decreases further toward the end of the gene, suggesting that there must be a rupture of the operon mRNA within this gene. This could be a result either of premature transcription termination or cleavage of the transcript by RNases. The results taken together indicate that the difference in the expression levels through the *C. crescentus isc-suf* operon could result from distinct rates of mRNA turnover, as described for the *E. coli isc* operon (Desnoyers et al., 2009).

### IscR may recognize two conserved DNA motifs

To determine the IscR binding sites in the whole *C. crescentus* genome, we constructed a strain encoding a chromosomally encoded 3xFLAG-tagged IscR to use in the ChIP-seq assay. This strain was grown in conditions of sufficient iron (cultures in PYE medium) or depleted of iron (cultures in PYE treated with 100 μM DP for 2 h). The addition of the 3xFLAG-tag did not alter the growth in these conditions (Supplementary Figure S6). The cell extracts were used for chromatin pulldown with an anti-FLAG resin and the DNA fragments isolated were identified by DNA sequencing (Supplementary Table S5).

**Figure 8:**
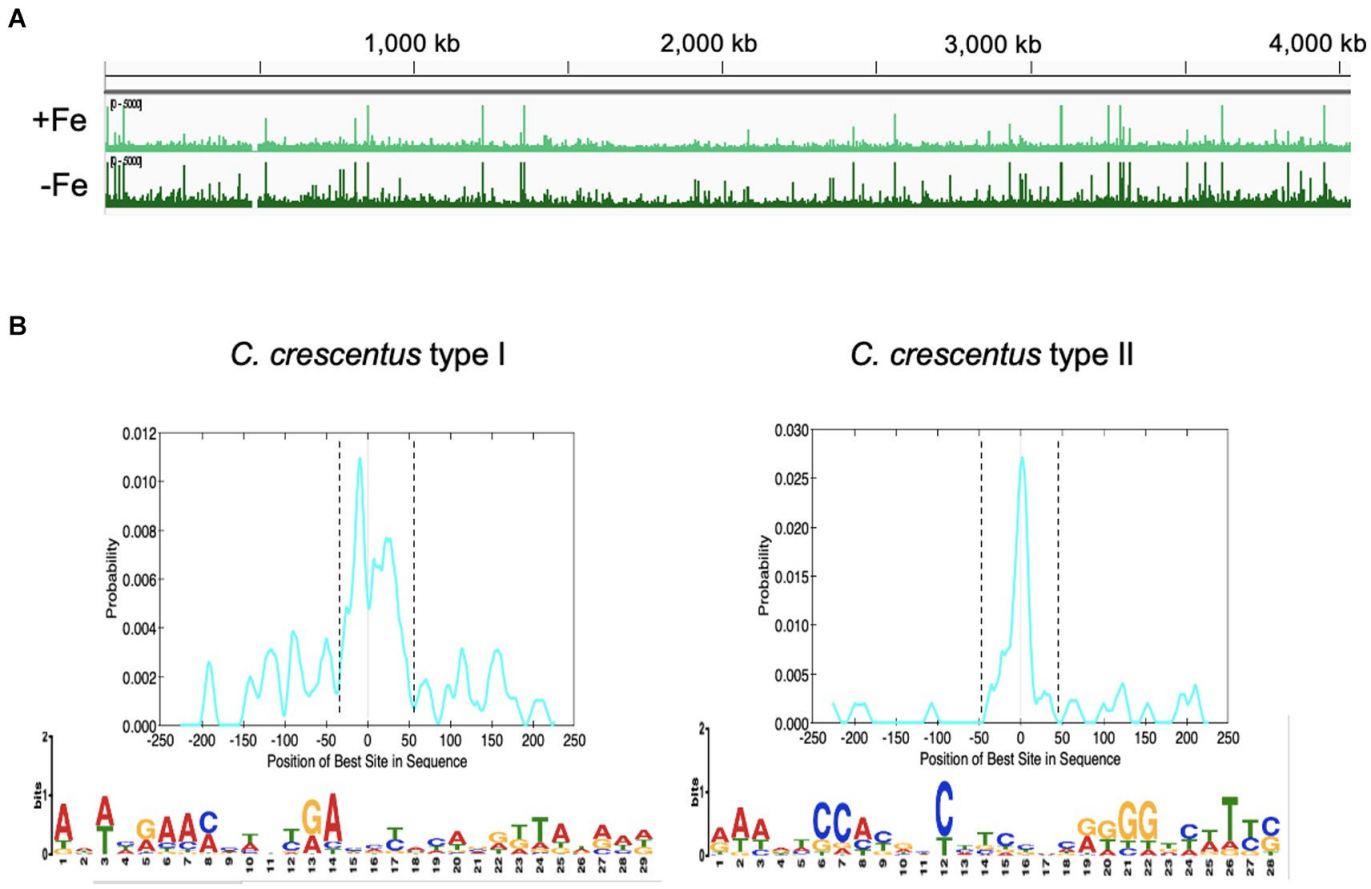
Mapping the IscR-binding sites in the *C. crescentus* genome. **(A)** Peaks of DNA reads distributed along the *C. crescentus* genome obtained in the ChIP-seq experiment from cultures in PYE medium (+Fe) or PYE + DP (−Fe). **(B)** Motifs enriched in the regions containing the IscR-binding sites. FASTA files of 500-pb sequences containing the peaks summits centralized were used as input in the MEME-ChIP (Motif Analysis of Large Nucleotide Datasets) program of the MEME suite (58). The localization of the motif in the input sequences was found by CentriMo in the MEME suite, within the dashed lines (graphs at the top). Best score (1.8e-15) Type I motif identified in 43/184 DNA regions that were not differentially bound by IscR in the presence of DP. Best score (6.3e-016) Type II motif identified in 36/52 DNA regions that were preferentially bound by IscR in the presence of DP. IscR binding motifs were generated using WebLogo.

The IscR regulator has been described to bind to distinct DNA sequences when in the apo-or holo-form, showing a [Fe-S] cluster-dependent DNA sequence discrimination (reviewed in Santos et al., 2015). The ChIP-seq experiment was carried out in two growth conditions: iron-sufficient (PYE medium) and iron-depleted (PYE + DP), which allows the identification of consensus sequences among IscR-binding peaks from each condition (Figure 8). We analyzed the results quantitatively by comparing the number of reads in the peaks between the cultures treated or not with DP, and only peaks with an average of the normalized reads higher than 1,000 were considered. In our analyses, 224 peaks were considered significant among replicates, with 90 differentially bound in each condition (88 more predominantly bound in DP, 2 more predominantly bound in PYE) and 133 with no difference in ligation between conditions. One hundred and twenty-six peaks localized in intergenic regions were considered to have a regulatory role.

While several peaks were present in both treatments, we considered peaks with more reads in PYE as sites predominantly occupied by holo-IscR and peaks with more reads in PYE+ DP as sites predominantly occupied by apo-IscR. However, although a 2 h treatment with DP has been shown to lead to iron depletion (da Silva Neto et al., 2009, 2013), this must be taken carefully since we cannot establish the real [2Fe-2S] occupation of IscR in our samples. We extracted 500 bp DNA sequences around the peak summit (250 bp on each side) and used them to search for conserved sequences with the MEME-ChIP tool (Machanick and Bailey, 2011). As shown in Figure 8B, we obtained two distinct motifs enriched at the peaks found for each of the conditions, which we named Type I (TypeI^Cc^) enriched in the iron-replete conditions, and Type II (TypeII^Cc^), enriched in the iron-depleted conditions. Only TypeII^Cc^ motif was found in peaks from both conditions, but with very few exceptions the peaks containing TypeI^Cc^ and TypeII^Cc^ were mutually exclusive in each gene. The TypeI^Cc^ motif (AWWHRAAMMWNYRAHMYMYAVRK TAWRWD) contains a widespread A-rich sequence as the Type 1 motif described for *E. coli*, which is remarkable given that the *C. crescentus* genome is 67% GC-rich. The TypeII^Cc^ motif (RAWDTCCAYRHCHTCBNCRKGGDYWTYS) is similar to the Type 2 motif described in *E. coli* (AWARCCCYTSnGTTTGMn GKKKTKWA) (Giel et al., 2006; Nesbit et al., 2009), with overall conservation of nucleotides but distinct spacing. We observed a conserved run of A-C in the first half motif followed by a run of G-T in the second half that indicates a symmetric or weak palindromic motif.

To confirm the ChIP-seq results *in vitro*, we tested IscR binding to some of the predicted DNA regions by electrophoretic mobility shift assay (EMSA) with an IscR-His purified from *E. coli* BL21(DE3). IscR was purified by heparin and ion exchange chromatography (IEX) since initial trials with nickel affinity showed high protein instability. The fractions from IEX presented a reddish color and when analyzed by UV–Vis spectroscopy demonstrated an absorption peak around 450 nm, suggesting the protein is in its holo state with [2Fe-2S] bound (Supplementary Figure S7).

**Figure 9:**
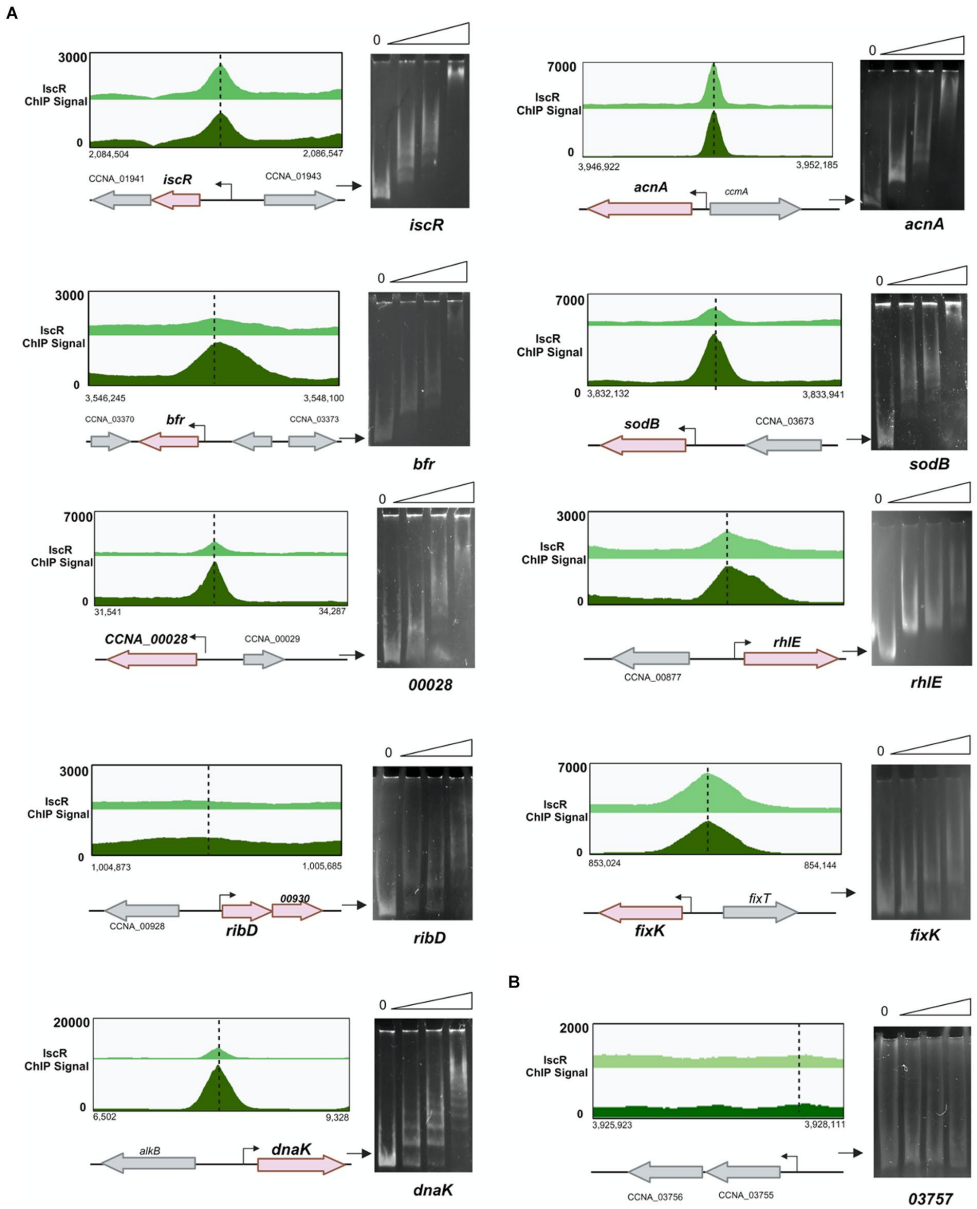
IscR binds to regulatory regions of several targets identified by ChIP-Seq. **(A)** Representative scheme of the peaks obtained for IscR-bound DNA reads in the ChIP-Seq experiment from cultures in PYE medium (light green) or PYE + DP (dark green) visualized by the Integrated Genome Browser. Electrophoresis Mobility Shift Assay (EMSA) experiments using DNA probes corresponding to each peak are shown at the right of the respective scheme. Probes were incubated with increasing concentrations (0–50–100-250 nM) of the purified IscR protein, as shown above each figure. Gels were stained with ethidium bromide. **(B)** The promoter region of gene CCNA_03757 was used as a negative control. The arrow indicates the free probes. Created in BioRender. Santos, N. (2024) BioRender.com/x20p050.

The EMSA showed that IscR-His bound to several DNA regions that showed peaks in the ChIP-seq experiment (Figure 9 and Supplementary Figures S8, S9). As the expression driven from the *iscR* promoter is increased in the Δ*iscR* strain, we carried out a DNA-protein interaction assay using fragment A of the *iscR* promoter (Figure 4A). A peak for IscR binding was identified in the ChIP-seq overlapping the-35 region, confirming the expression results, and the EMSA results also confirmed the IscR binding (Figure 9). Although a second peak for IscR binding was identified at the end of the *sufB* gene, it is distant from the promoter of the sRNA R0151 encoded in the opposite strand from *sufB*, and likely does not regulate it. Moreover, there is still no evidence that it has any role in the expression of the downstream genes.

We identified several relevant genes involved in iron homeostasis and other stress responses as direct targets for IscR binding (Figure 9). The *in vitro* binding of IscR was confirmed for the aconitase (*acn*) regulatory region where the peak of IscR binding identified in ChIP-seq was located overlapping the-35 region in the promoter. The gene encoding the bacterioferritin (*bfr*) has a putative IscR target sequence upstream of its promoter, as well as the *sodB* gene encoding the iron-manganese superoxide dismutase, CCNA_00028 for the TonB-dependent putative iron transport, the *rhlE* gene encoding a DEAD-box RNA helicase, the operon for the synthesis of riboflavin and the *dnaK* gene (Figure 9 and Supplementary Figure S9). Other targets confirmed include the regulatory regions of the genes encoding the Hfq and RpoH (sigma32) protein (Supplementary Figures S8, S9).

A very interesting result was the IscR binding to the *fixK-fixT* intergenic region (Figure 9). A sequence with good similarity to TypeII^Cc^ motif was found overlapping the putative promoter and TSS of *fixK*, indicating that IscR represses *fixK* transcription. Although the *fixK* gene was not differentially expressed in the RNAseq most of the genes belonging to the *fixK* regulon were, as an indirect confirmation that the *fixK* gene is more expressed in the *iscR* mutant (Crosson et al., 2005). The patterns of expression of the operons for the respiratory terminal oxidases *ccoNOPQ-fixGHI*, *cydCD*, *cydAB*, and *qoxABCD* and the *fixL* gene were consistent with the higher expression of FixK in the cell. Taken together, the results indicate that IscR binds as an apo-enzyme, repressing transcription of *fixK* at low iron.

The genes differentially expressed in the mutant as determined by RNA-seq can be either directly or indirectly regulated by IscR. Twenty-two differentially expressed genes with an IscR binding site upstream identified by the ChIP-seq were considered as being directly regulated. A comparison between the IscR regulon and the Fur regulon (da Silva Neto et al., 2013; Leaden et al., 2018) showed that 63 genes are shared by both regulators (Figure 6C and Supplementary Table S6). We found several peaks for IscR binding upstream of regulators that could in turn affect the expression of specific sets of genes, as described for FixK above. Among those, it is of notice the stationary phase response regulator SpdR (Da Silva et al., 2016), the response regulator TacA (Marques et al., 1997),and the cell cycle regulator CtrA (Quon et al., 1996), although the relevance of these findings remains to be established.

## Discussion

In this work, we have characterized the IscR regulon, to evaluate the role of IscR in controlling the expression of genes encoding proteins using iron or [Fe-S] clusters as cofactors, as well as others important to iron homeostasis. The important role of [Fe-S] clusters as prosthetic groups in many enzymes has long been recognized, and more recently it was proposed that Fur also binds a [2Fe-2S] cluster (Fontenot et al., 2020; Fontenot and Ding, 2023), emphasizing the crosstalk of signals and regulators that control the actual iron concentration within the cell. While the most studied regulator of this process is Fur, other players have an important role in defining gene expression, both at the transcriptional and post-transcriptional levels. Recently, an sRNA induced in response to iron starvation was described that regulates a small set of genes in *C. crescentus*, mainly for outer membrane transporters, in an Hfq-dependent way (Vogt et al., 2024), but most of the iron-responsive regulation is likely performed by proteins.

Previously, it was found that the *isc-suf* operon is upregulated at low iron conditions, in a Fur-independent way (da Silva Neto et al., 2013). The results from the beta-galactosidase assays of an P*iscR/lacZ* fusion (Figure 4) showed that the levels of expression in the presence of DP are the same as those in the *iscR* mutant, indicating that IscR is repressing transcription initiation of the *isc-suf* operon in the presence of iron (as a holo-IscR protein). In fact, a peak for IscR binding was localized overlapping the-35 region of the promoter and IscR bound with high affinity to the *isc* regulatory region (Figure 9); this region contains a sequence with low similarity to the predicted TypeII^Cc^ motif (Supplementary Figure S4). Interestingly, in the presence of DP we observed a further increase in expression when sequences upstream from the promoter were present in the transcriptional fusion (Figure 4) suggesting that there might be a second layer of regulation.

Strikingly, unlike the *suf* operon in *E. coli*, *C. crescentus isc-suf* operon is not induced in oxidative stress by H_2_O_2_ or paraquat, and is not regulated by OxyR (Figure 5). In contrast, it was previously shown in *E. coli* that oxidative stress induction of *suf* expression is mediated by OxyR (Lee et al., 2004; Mettert and Kiley, 2014). A model comparing regulation in both bacteria is shown in Supplementary Figure S5C. A recent work showed that in *E. coli* the small regulatory RNAs OxyS and FnrS have opposite effects on *iscR* expression (and consequently the *suf* operon) by interaction with its 5’-UTR, where the *iscR* induction by oxidative stress in aerobic conditions required OxyS (Baussier et al., 2023). The existence of a single operon in Alphaproteobacteria for the synthesis of [Fe-S] clusters suggests that its expression must be efficient in a large set of conditions. Moreover, the strict aerobic lifestyle of *C. crescentus* is a possible reason for the *isc*-*suf* expression not being affected when the cells are subject to oxidative stress, since the Suf proteins must be functional at high oxygen concentration.

In *E. coli*, the levels of expression of the *iscRSUA-fdx-hscBA-fdx-iscX* operon are regulated by the sRNA RyhB that leads to the degradation of the mRNA downstream of *iscR* (Desnoyers et al., 2009; Prévost et al., 2011). Measuring the presence of the mRNA by RT-qPCR using probes for distinct regions of the *isc-suf* operon, we observed that the levels of mRNA differ between the beginning and the end of the *iscS* gene (Supplementary Figure S5B), indicating that the *iscS* ORF could be the site for post-transcriptional regulation. Within the *sufB* gene, there is a gene coding for the antisense sRNA CCNA_R0151 that has not been characterized but could have a role in regulating the downstream genes. Another result of note was that a peak for IscR binding was identified at the end of *sufB* and was more predominant in iron scarce conditions. Although the relevance of these findings is still unknown, they might indicate that the *suf* genes could also be regulated separately in response to other environmental conditions.

We determined the *C. crescentus* IscR regulon by comparing global gene expression in the *iscR* mutant with the wt and identifying the IscR binding sites in iron depletion or sufficiency conditions. Analysis of conserved sequences present in the ChIP-seq peaks regions identified two conserved motifs. TypeII^Cc^ motif shares good similarity with the *E. coli* type 2 motif, suggesting that they are conserved between these two genera. In fact, *C. crescentus* IscR shares all the critical residues as *E. coli* IscR for DNA binding to the *hya* promoter (a type 2 motif): S40, Y41, E43, Q44 and R59 (in *C. crescentus* IscR: S43, Y44, E46, Q47 and R62) (Figure 2A) (Rajagopalan et al., 2013). Although we cannot establish in this work whether IscR is binding in the apo-or holo-form, the TypeII^Cc^ motifs are predominant in peaks from the DP-treated cells, suggesting it is preferentially occupied by apo-IscR. Structural studies of *E. coli* IscR showed that the ligation of a [2Fe-2S] cluster to one monomer of IscR causes a reorganization of the DNA-binding domain so that the dimer switches from exclusively binding to the type 2 motif to binding to both type 1 and type 2 motifs (Rajagopalan et al., 2013), and this is affected by the O_2_ availability (Giel et al., 2006; Nesbit et al., 2009). If this is also occurring in *C. crescentus* IscR, the combined effect of the [Fe-S] clusters and O_2_ concentrations could fine-tune modulate gene expression over a wide range of conditions.

Besides regulating the iron–sulfur clusters biosynthesis genes, several genes belonging to the IscR regulon in *C. crescentus* are also regulated by IscR in other bacteria. For instance, processes such as iron transport through the outer membrane (CCNA_00028, CCNA_02370, CCNA_02743, CCNA_03444, and CCNA_03574) and oxidative stress response (*ahpCF*, *sodB*) (Lim et al., 2014; Schwiesow et al., 2018). Interestingly, IscR-regulated genes coding for iron permeases (*feoAB* in *Rhodobacter sphaeroides*, *feoB* in *Yersinia*) or oxidative stress response (*katAG* in *Y. pseudotuberculosis*) in other bacteria were not differentially expressed in the *C. crescentus iscR* mutant (Remes et al., 2014; Balderas et al., 2021).

**Figure 10:**
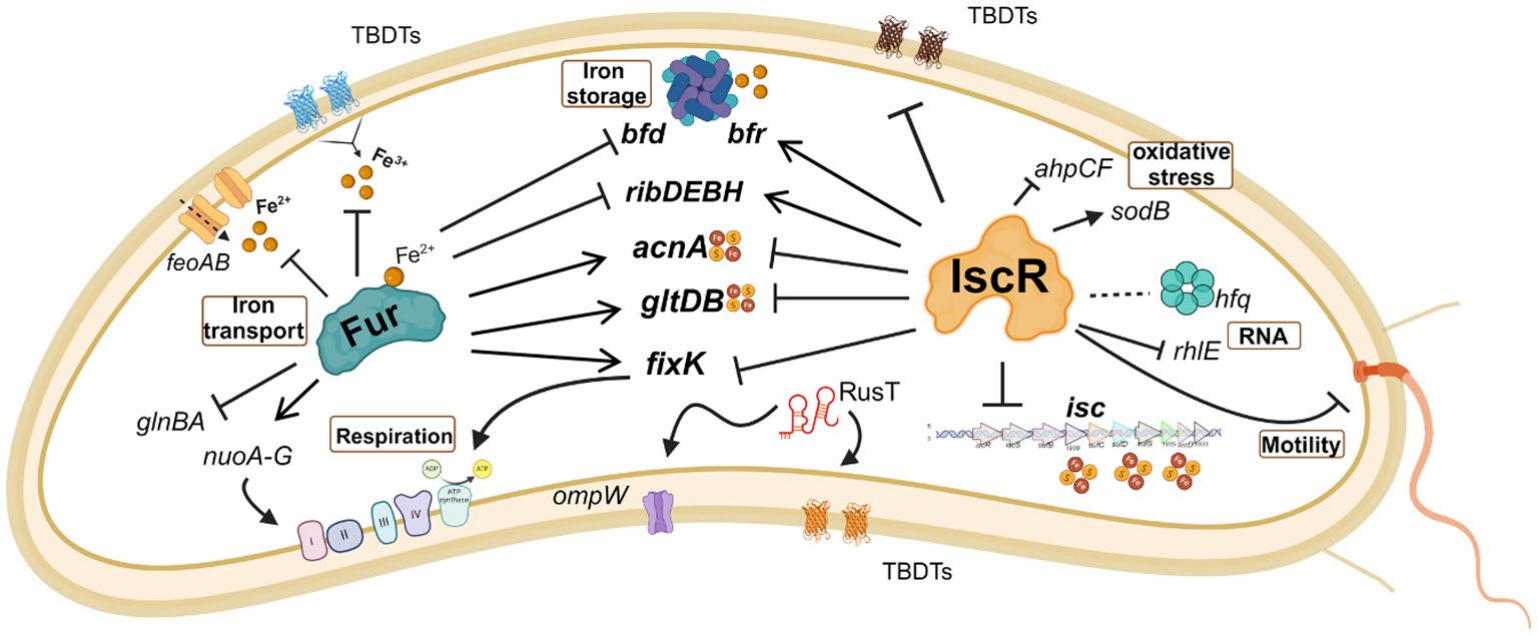
Schematic representation of some of the processes regulated by IscR and/or FUR. The effect of the regulator in gene expression is indicated as activation (arrow heads) or repression (blunt heads). Traced lines indicate that the result of the regulation (activation or repression) is unknown. The red and yellow circles symbolize the Fe-S clusters. Note that although IscR is showed without the Fe-S group, it may bind as a holo-protein to some operators. Created in BioRender. Santos, N. (2024) BioRender.com/o32b986.

A summary of the most important effects of IscR and Fur in *C. crescentus* iron homeostasis is shown in Figure 10. The riboflavin biosynthesis operon was downregulated in the *iscR* mutant, indicating that it is activated by IscR (Figure 6A and Supplementary Table S4), which binds to the regulatory region upstream of the *rib* operon (Supplementary Figure S10). The most common regulation of riboflavin synthesis genes among bacteria occurs post-transcriptionally through an FMN riboswitch (Winkler et al., 2002; García-Angulo, 2017; Vikram et al., 2022). In *C. crescentus* strains the riboswitch RFN element is not present upstream of *ribD* (Vitreschak et al., 2002), implicating that the regulation of riboflavin biosynthesis is exclusively transcriptional and mediated by Fur and IscR. Fur represses transcription of the *rib* operon in the presence of iron (da Silva Neto et al., 2009, 2013) and there is a putative Fur binding site between the putative-35 and-10 regions. IscR activates transcription, likely in the apo-form, and a putative IscR binding motif 1 is found upstream of the-35 element.

Interestingly, several IscR-binding sites identified in the ChIP-seq experiment were in regulatory regions where other transcriptional regulators bind (Supplementary Figure S10). The *acn* gene is downregulated in the presence of DP (Leaden et al., 2018), directly activated by Fe-Fur (da Silva Neto et al., 2009) and there is an IscR binding site immediately downstream of the Fur operator, overlapping the-35 region (Supplementary Figure S10A). The *bfd* gene, encoding a [4Fe-4S] containing bacterioferritin-associated ferredoxin, is repressed by Fe-Fur (da Silva Neto et al., 2013), while an IscR binding site is found around position-100 in the bacterioferritin *bfr* regulatory region, likely activating the gene. We identified a peak for IscR binding in the intergenic region between the divergent genes *alkB* and *dnaK*. Previous work has shown that the *rpoH* gene encoding σ^32^ and some genes belonging to its regulon are upregulated in iron deficiency (da Silva Neto et al., 2013; Leaden et al., 2018), but not the *dnaKJ* operon. Interestingly, the IscR predicted binding site overlaps the-35 region of the σ^32^ promoter of *dnaK* (Supplementary Figure S10A), suggesting that *dnaK* transcription could be more efficient at low iron and that IscR could have a negative effect in iron-replete conditions.

In some genes the effect is antagonistic, probably by IscR binding interfering with the binding of other regulators (Supplementary Figure S10B). The *ahpCF* operon encoding a NADH-dependent peroxiredoxin important for oxidative stress defense is directly activated by OxyR (Italiani et al., 2011) and it is upregulated in the *iscR* mutant (Supplementary Table S4). Although the putative IscR binding site is located upstream of the OxyR operator (centered around position-100), we speculate that it could interfere with OxyR binding causing a lack of activation. The same could be observed for the *gltDB* operon, encoding the glutamate synthase, where binding of IscR could impair the activation by Fe-Fur. In fact, several cellular processes are regulated by both IscR and Fur (Figure 10), showing that sensing and responding to iron levels requires both regulators.

We observed in the EMSA that IscR binds to the promoter region of the *fixK* gene, in a position that would repress expression (Supplementary Figure S10B). FixK coordinates the expression of the operons for respiratory terminal oxidases according to oxygen concentration (Crosson et al., 2005). Several of these enzymes use either Fe-Heme or [Fe-S] clusters as a cofactor, and their expression must be coordinately regulated in response to the levels of [Fe-S] clusters in the cell. In fact, IscR binding sites were predicted for the *cyaA-cyaD* and for *coxB*-CCNA_03519 divergent intergenic regions. It was previously described in *C. crescentus* that *fixK* was downregulated in iron starvation and in the *fur* mutant (da Silva Neto et al., 2013), but no canonical Fur binding site was found in the *fixK* regulatory region, indicating this might be an indirect effect. This indicates that IscR preferentially binds to the operator in the apo-form, explaining the downregulation of *fixK* under iron depleted conditions.

The FixK regulation by IscR is an important point of intersection between iron homeostasis and oxygen concentration and may be relevant to the diversity of environments faced by this free-living bacterium. The Fix signaling module consists of the two-component phosphorylation system FixL-FixJ, and signal transduction is inhibited by the FixT protein when bound to a [Fe-S] cluster (Stein et al., 2020). FixK is the transcriptional activator of these three genes, and its repression by IscR in conditions where [Fe-S] groups are damaged or insufficient ensures that the respiratory components synthesis respond to the redox cell state.

Here we showed that *C. crescentus* exhibits an intricate regulation of iron-dependent processes for central cellular functions, allowing a fast adaptation to the bacterium’s natural habitat of low nutritional availability and stress conditions. The results obtained have shed light on how the transcriptional regulator IscR senses the [Fe-S] pool and might act in concert with Fur to maintain the balance of gene expression. The combined regulation by both factors on several genes allows a fine-tuning of expression levels in response to iron and redox state, which is relevant for basal metabolism performance in a timely fashion. While IscR and Fur are the key regulators of iron homeostasis in *C. crescentus*, other players could still be participating in this network, and this is currently under investigation.

## Data Availability

The datasets presented in this study can be found in online repositories. The names of the repository/repositories and accession number(s) can be found at: https://www.ncbi.nlm.nih.gov/, PRJNA1124373, https://www.ncbi.nlm.nih.gov/, SRP136695, https://www.ncbi.nlm.nih.gov/geo/, GSE45653.

## Footnotes

^1^https://github.com/alanlorenzetti/frtc
