# Supplementary material for: Mapping the IscR regulon sheds light on the regulation of iron homeostasis in Caulobacter
Source: Front Microbiol. 2024 Sep 30;15:1463854. doi: 10.3389/fmicb.2024.1463854 (PMC11475020; doi:10.3389/fmicb.2024.1463854)
Supplement: Supplementary file 1 [file Data_Sheet_1.zip › Supplementary Tables 1, 2 and 3.pdf]

Supplemental material for "Mapping the IscR regulon sheds light on the regulation of iron homeostasis in *Caulobacter*"

Naara M. dos Santos, Beatriz A. Picinato, Lucas S. Santos, Hugo L. de Araújo, Andrea Balan, Tie Koide, Marilis V. Marques

Table S1 – *C. crescentus* and *E. coli* strains

Strain	Description	Reference
C. crescentus		
NA1000	Synchronizable parental strain	Evinger; Agabian, 1977
Δ*fur*	NA1000 (Δ*fur*)	Silva Neto et al., 2009
Δ*oxyR*	NA1000 (Δ*oxyR*)	Italiani et al., 2011
Δ*iscR*	NA1000 (Δ*iscR*)	This work
IscR-FLAG	NA1000 encoding an IscR protein with a FLAG tag at the C-terminus	This work
E. coli		
DH10β	*F-mcrAΔ(mrr-hsdRMS-mcBC) Φ80lacZΔM15ΔlazX74recA end A1*	Hanahan, 1983
S17-1	294:RP4-2(Tc:: Mu) (Km::Tn7)	Simon et al.,1983
BL21 (DE3)	*F^- ompT gal dcm lon hsdS$_B$(r_B^- m_B^-) λ(DE3 [lacI lacUV5-T7 gene 1 ind1 sam7 nin5])*	Novagen

Table S2 – Plasmids used in this work

Plasmid	Caracteristics	Reference
pNPTS138	Replicon ColE1, *oriT, npt* (Km^r), *sacB.*	M.R.K. Alley

pRKlacZ290	Vector with promoterless *lacZ*, Tet[r]	Gober and Shapiro, 1992
Zero Blunt® PCR Cloning	Cloning vector	Ivitrogen
pET28a+	Expression vector with histidine-tag fusion	Novagen
pNPTΔ*iscR*	pNPTS138 with *iscR* flanking regions	This work
pNPT-IscR-FLAG	pNPTS138 with *iscR* flanking regions and codons for 3xFLAG added to the end of the *iscR* gene	This work
p*lacZ*FragA	pRKlacZ290 with Fragment A of *iscR* promoter in fusion to *lacZ*	This work
p*lacZ*FragB	pRKlacZ290 with Fragment B of *iscR* promoter in fusion to *lacZ*	This work
p*lacZ*FragC	pRKlacZ290 with Fragment C of *iscR* promoter in fusion to *lacZ*	This work

Table S3 – Primers used in this work

**Name**	**Sequence (5'-3')**
01942IF-1	GATATCTGGATCCACGAATTCCGAGATAGTCGCTGACGC
01942IF-2	TTCACTCGGCGCGCATCAGCCTTACCCC
01942IF-3	GCTGATGCGCGCCGAGTGAACGCTTCCCG
01942IF-4	ACGGCCGAAGCTAGCGAATTCAGGCGTCGCGCCATTGGG
42COMPF	AAGCTTGACGGCGGAGCCCGGCTTTTTAG
42COMPR	GGATCCGCGGCCGGCGGCATGGACCGATG
pLacZFB	GAATTCCAGGCATATCGTCCCCTTCAGC
pLacZFC	GAATTCTTGACCGCCGCGCTTGGTCTTC
pLacZFA	GAATTCGACCCTCGATCCGGCCGGAC
pLacZ2R	GAATTCCTTGGCCAGGCGATAGCCGC
RT 42 Fw	CGGTGCATGACGCATAATCTC
RT 41 Rv	TTATCCACAGGCAGGGCCTC
q/RT 41 Fw	GGCTCCGAAGTGCAGGTCA
RT 40 Rv	GCGTAGTAATAGGTGTCCTGGT
RT 39 Fw	CCGTCTGAGGATCTTTCGC
RT 38 Rv	GTCGATCTTCAGAGACGCGG

RT 37 Rv	TTGACGACGATGACGTGGCT
RT 36 Fw	GAATGGCTGAACGGTCTGGAT
RT 36 Rv	CGACAGGCCGAACGAACT
RT 35 Fw	AAGGACGCGGTCATGGAGAT
RT 35 Rv	AGATCAGGCCCAGCTCATAG
RT 34 Fw	TGGACTGATGCTGGCGAAAA
RT 34 Rv	GGATCGTCACGCTCTCACC
RT 33 Rv	CAGACCTCGACCGGACACT
qRT_01942 Fw	CTGCAACTTCACCAAGGGTC
qRT_01942 Rv	AGATAGCCGTGGATCTGCCG
qRT_01941 Fw	GGTTCGGTCACCTTCGTCAG
qRT_01941 Rv	CATGCTCGATGGCGGAAATG
q/RT 41 Fw	GGCTCCGAAGTGCAGGTCA
qRT01941 Rv Final	GGGCCAGATCTGCCCGGC
qRT_01940 Fw	GAACTGGTCCGCCAGTATCT
qRT_01940 Rv	GGGACGTAGACAAAGGAGCC
qRT_01939 Fw	GCTATGAGGTCACCGAAGGC
qRT_01938 Rv	GCGGATATTGGAACGACAGGA
qRT_01937 Fw	GCTGGACGACGAAGTGTTGT
qRT_01937 Rv	GCGATCCGCTCGATCACTT
qRT_01934 Fw	TATCCTGATCGAACCCAAGGC
qRT_01934 Rv	CGGTTTCGTTCGGATTGTGG
qRTCCNA_01604 Fw	AAGGACAAGACGGTTCAGGG
qRTCCNA_01604 Rv	CTGATATTGCCGGTCAGGGG
qRTCCNA_03446 Fw	ATCTCTTCACCTCCGGCTCA
qRTCCNA_03446 Rv	CTCCAGGGCATCCATTCCAG
qRTCCNA_01852 Fw	ATTCTGGGCCTGTCCTTTGG
qRTCCNA_01852 Rv	GATCAGCCACGAGAGATCCG
qRTCCNA_02235 Fw	ATCCTACATTCTGACCGGCG
qRTCCNA_02235 Rv	CGACTGGACGTCGATACTGG
qRTCCNA_00866 Fw	GGCCTGTGGTCGCAGTATCT
qRTCCNA_00866 Rv	CCAGTGGCTGTCATAGCGGA
qRTCCNA_2370 Fw	GACGGATCCGAACAACTCGT

qRTCCNA_2370 Rv	ACAGCTCCTTGTGGTACACG
qRTCCNA_2369 Fw	CTGGATGGCGTCTTTACCGA
qRTCCNA_2369 Rv	CCAGAACTCCGGGTTCACAT
qRTCCNA_2689 Fw	GCTTCGGCTTTCCCTTCATC
qRTCCNA_2689 Rv	CAGGCGAAGCTTGGAGATCA
IscR Fw	GATATCTGGATCCACGAATTCCGAGATAGTCGCTGACGC
IscR Rv	ACGGCCGAAGCTAGCGAATTCAGGCGTCGCGCCATTGGG
1.5 Flag IscR Fw	GTCCTTGTAGTCGCCGTCGTGGTCCTTGTAGTCCTCGGCCGCGATCTC CATCGGGC
1.5 Flag IscR Rv	CACGACATCGACTACAAGGACGACGACGACAAGTGAACGCTTCCCG ACCCTCGGTC
pETIscR1	GGAGATATACCATGGCTATGCGCCTGAGCACCAAA
pETIscR2	GTGCGGCCGCAAGCTTCTCGGCCGCGATCTCCAT
*acn*Shift Fw	GAAAACAGCGTTCGCTCACC
*acn*Shift Rv	AAACCACTCCCGGAATCACG
CCNA_00028Shift Fw	TGGCAGATTGGAGTTAGGCG
CCNA_00028Shift Rv	TCGGGTTACGCATAGTGGTC
CCNA_00265Shift Fw	CTGGTTGTCGGAAGGAACGA
CCNA_00265Shift Rv	CTGATCGTCAGGAGCTTGGG
*sodB*ShiftFw	CTTTCCTGGATCCCAAGCCG
*sodB*ShiftRv	GTTCCCATGAAGGGAGGAGC
*lon*Shift Fw	AAGCCTCCTGCAGCGATTAG
*lon*Shift Rv	GGTCAAGTTGAGCGCCCTAT
*bfd*ShiftFw	CATCTTCCGTCACAAGGGCT
*bfd*ShiftRv	CCTTAGGCGTCGAGGATTCG
*dnaK*Fw	TTGGCGATCATCTGGAGCCC
*dnaK*Rv	AATCAAACGGGCGGAATGCG
*ribB*Fw	CGCGGGGAAACTTGCCATC
*ribB*Rv	TCGGAAAAGCCCTGGATCACG
*hfq*Fw	CCCGTGAGGCTTTCATCACC
*hfq*Rv	TCATGCTAGAGGATTTTTCGCCG

References

da Silva Neto JF, Braz VS, Italiani VC, Marques MV. Fur controls iron homeostasis and oxidative stress defense in the oligotrophic alpha-proteobacterium *Caulobacter crescentus*. Nucleic Acids Res. 2009 Aug;37(14):4812-25.

Evinger M, Agabian N. 1977. Envelope associated nucleoid from *Caulobacter crescentus* stalked and swarmer cells. J Bacteriol 132:294–301.

Gober JW & Shapiro L (1992) A Developmentally Regulated *Caulobacter* Flagellar Promoter is Activated by 3' Enhancer and IHF Binding Elements. Mol Biol Cell 3: 913–926

Hanahan, D. Studies on transformation of *Escherichia coli* with plasmids. Journal of Molecular Biology, v. 166, n. 4, p. 557–580, 1983.

Italiani VCS, Da Silva Neto JF, Braz VS & Marques M V. (2011) Regulation of catalase-peroxidase KatG is OxyR dependent and fur independent in *Caulobacter crescentus*. J Bacteriol 193: 1734–1744

Simon R, Priefer U, Pühler A. 1983. A broad host range mobilization system for in vivo genetic engineering: transposon mutagenesis in gram negative bacteria. Biotechnology, 1: 784-791.
