# Supplementary material for: Mapping the IscR regulon sheds light on the regulation of iron homeostasis in Caulobacter
Source: Front Microbiol. 2024 Sep 30;15:1463854. doi: 10.3389/fmicb.2024.1463854 (PMC11475020; doi:10.3389/fmicb.2024.1463854)
Supplement: Supplementary file 1 [file Data_Sheet_1.zip › Supplemental figures New.pdf]

Supplementary Material

dos Santos et al., Mapping the IscR regulon sheds light on the regulation of iron homeostasis in Caulobacter

corresponding author: Marilis Marques
Email: mvmarque@usp.br

This PDF file includes:

Figures S1 to S10
References

Supplementary Figure S1

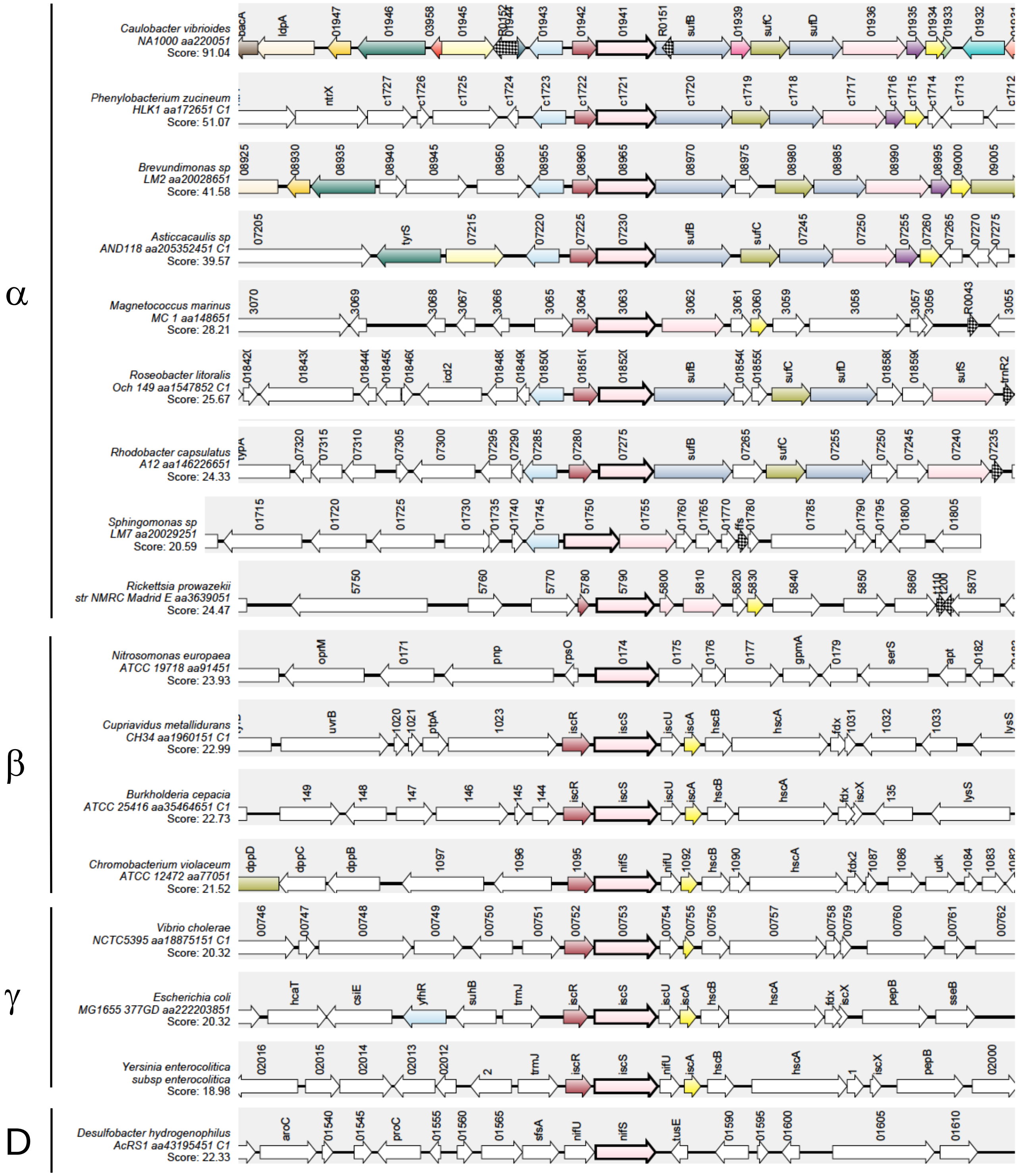

Figure S1. Synteny of the *iscS* gene among selected bacteria. The genes flanking the *iscS* gene in each chromosome are colored to indicate those encoding orthologous proteins. Synteny analysis was done based on *C. crescentus* IscS (CCNA_01941) with the SyntTax program (Oberto, 2013). Three groups of Pseudomonadota (alpha, beta and gamma proteobacteria) are indicated by the respective Greek letters. Desulfobacter (D) has a NifS ortholog. The scores refer to similarity to *C. crescentus* IscS by TBLASTN.

Supplementary Figure S2

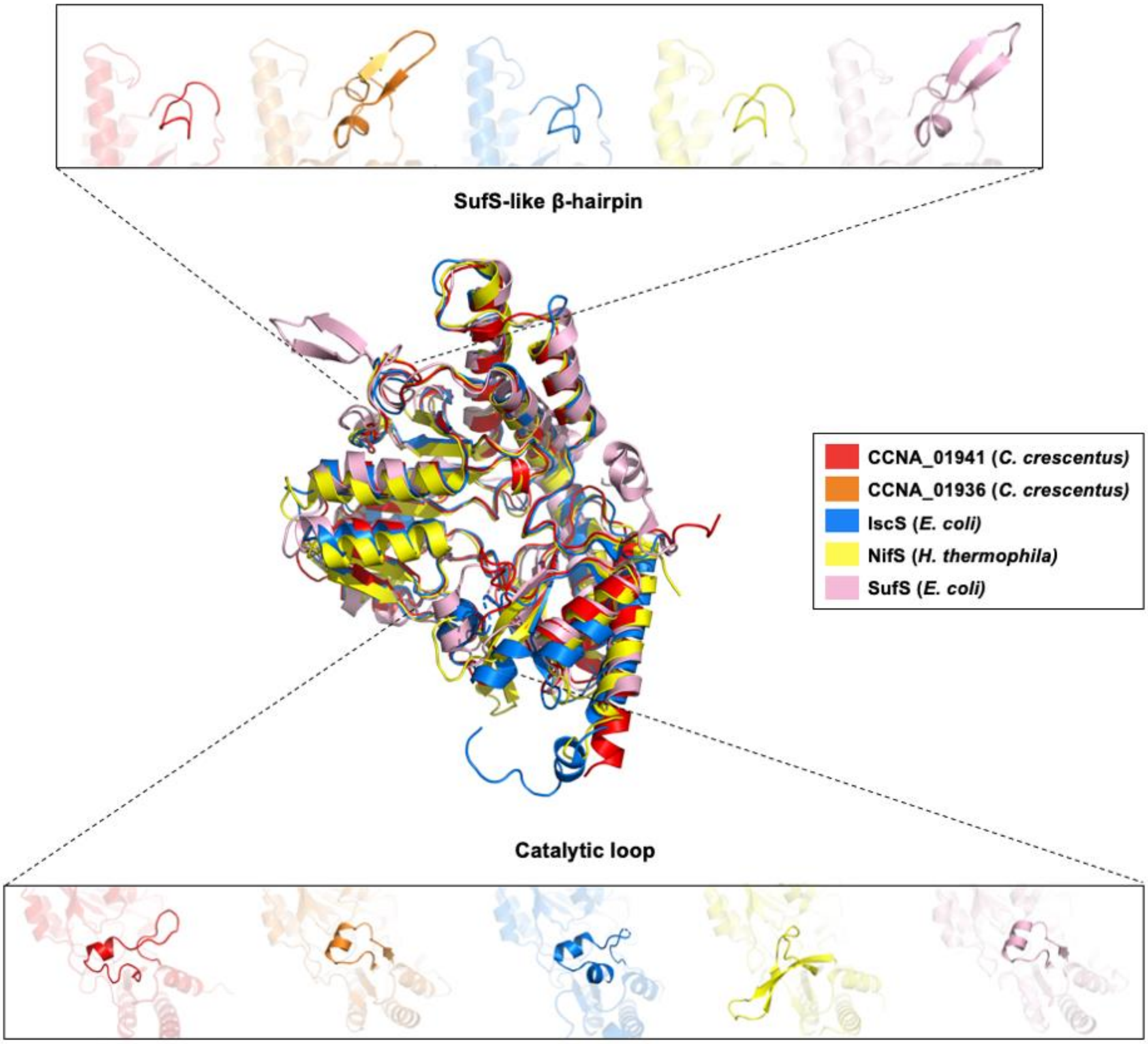

Figure S2. Protein structure comparison of monomers of the cysteine desulfurase family. CCNA_01936 and CCNA_01941 were modeled by AlphaFold2 and superposed against *E. coli* SufS (PDB: 6O10), IscS (PDB: 3LVM), and *H. thermophila* NifS (PDB: 5ZSP). Local structural features from the catalytic loop region and in the vicinity of the dimerization interface suggest the characterization of CCNA_01936 and CCNA_01941 as SufS-like and IscS-like, respectively.

Supplementary Figure S3

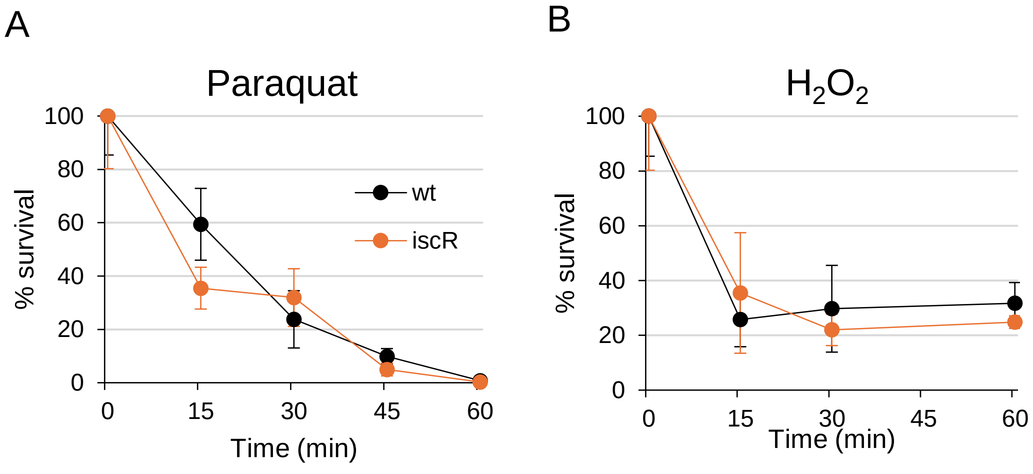

Figure S3. Phenotypic analysis of the *iscR* mutant. Survival test of the NA1000 (wt) and Δ*iscR* strains after incubation with 1 mM Paraquat (A) or 1 mM H_2O_2 (B). Percentage of survival measured as CFU counts was calculated relative to time zero (no stressor). The graphs are the average of three independent tests.

Supplementary Figure S4

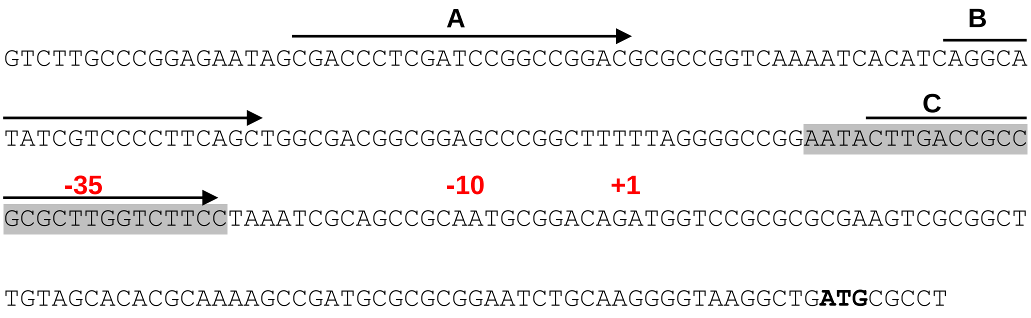

Figure S4. DNA sequence of the *isc* regulatory region. The position of the forward primers used to generate fragments A, B and C for cloning into vector pRK*lacZ*290 are indicated by arrows. The position of the promoter -35 and -10 regions and the transcription start site (+1) are indicated by red numbers above the sequence. A sequence corresponding to the IscR binding peak determined by ChIP-seq is shaded. The start codon for IscR is shown in boldface.

Supplementary Figure S5

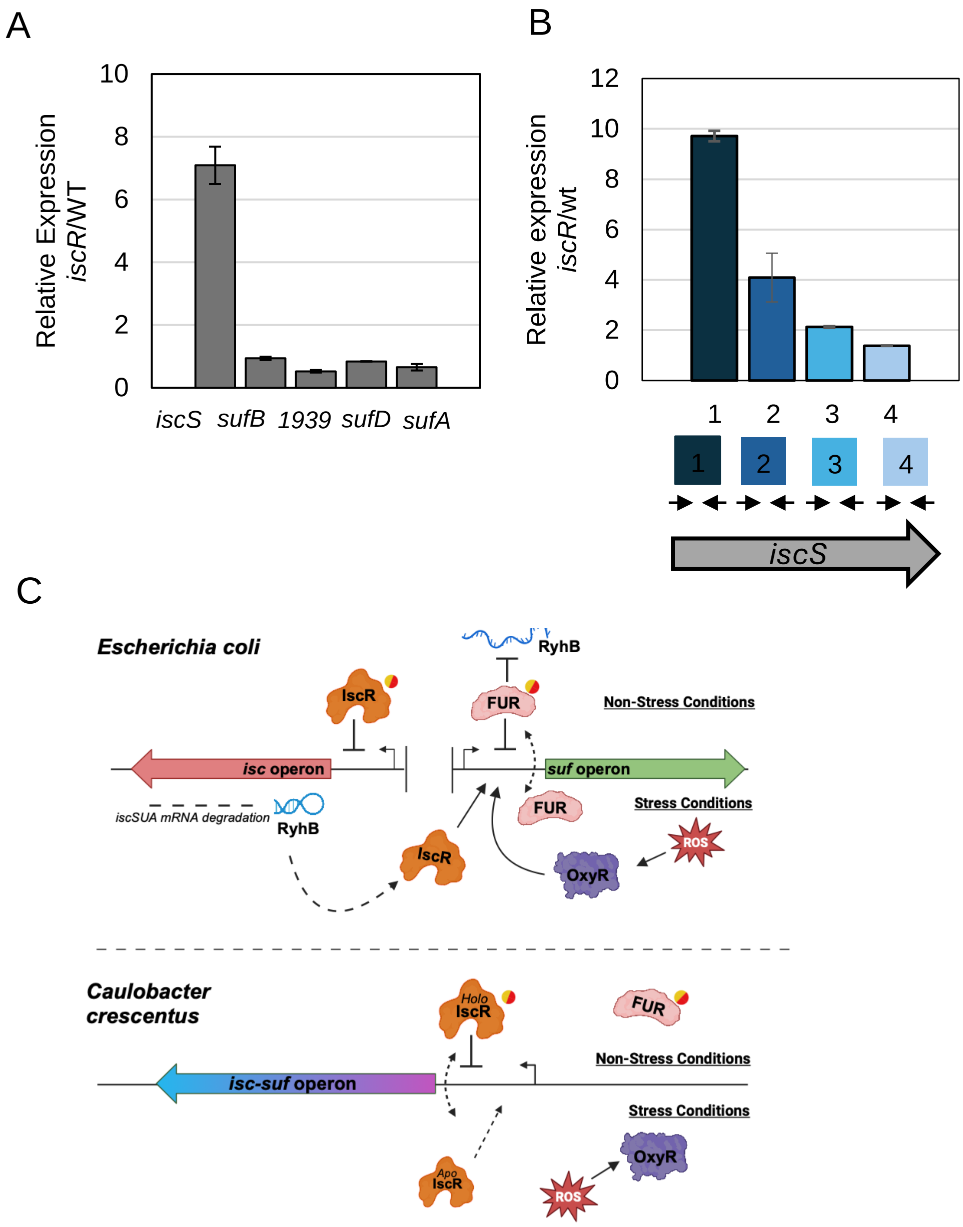

Figure S5. Regulation of individual genes from the *isc-suf* operon in the *iscR* mutant. A. Relative expression of genes from the *isc-suf* operon in the *iscR* mutant vs. wt strain. B. Expression of *iscS* was evaluated using primer pairs at the beginning (1), middle (2, 3) or at the end (4) of the *iscS* region of the mRNA, in the *iscR* mutant relative to wt. Expression was determined by RT-qPCR and the value for each gene was normalized to that at the wt strain. C. Schematic view of the regulation of the *isc/suf* operon from *C. crescentus* compared to the *isc* and *suf* operons in *E. coli*. Created in BioRender. Santos, N. (2024) BioRender.com/k05u539.

Supplementary Figure S6

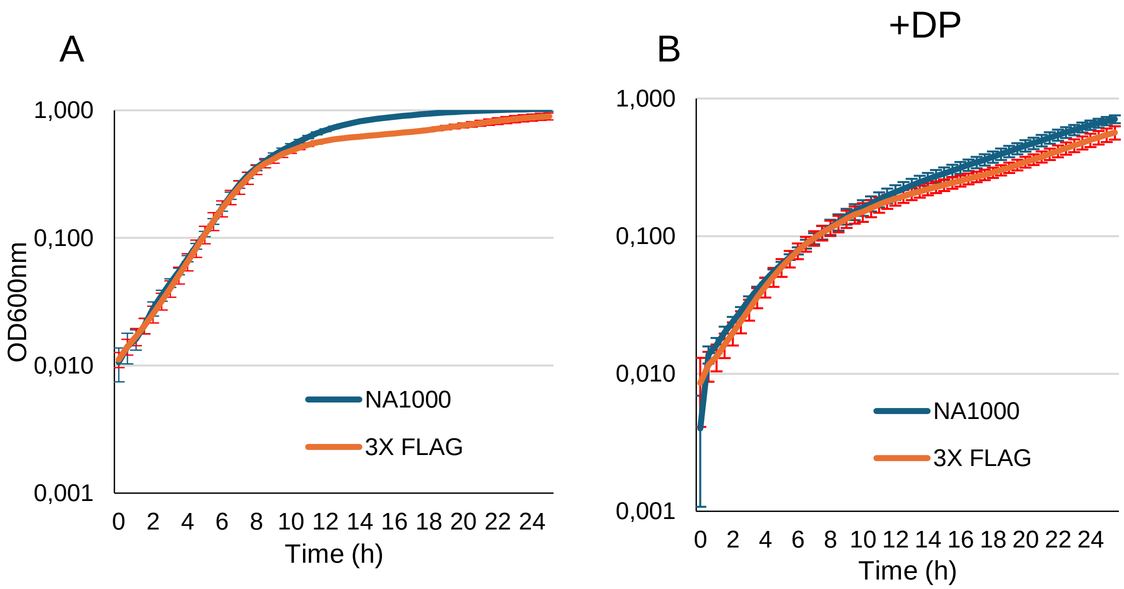

Figure S6. Growth of the wt and FLAG-IscR strain. The cultures were grown in PYE (A) or in PYE with the addition of 100 µM DP (B) at 30°C with agitation. Growth was assessed by measuring the OD_{600nm} of six independent biological replicas.

Supplementary Figure S7

A

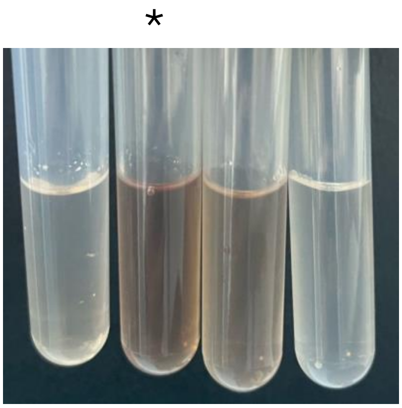

B

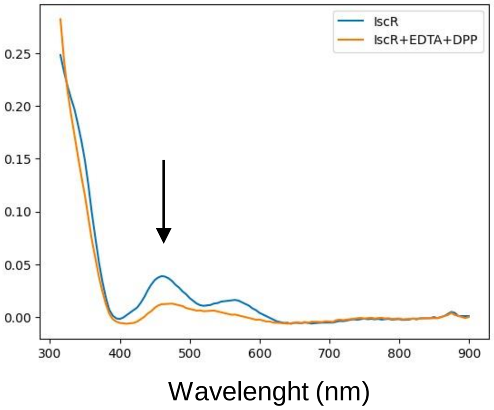

Fig. S7. Purification of the IscR-His protein. A. Fractions from an ion-exchange chromatography column. Total protein from *C. crescentus* NA1000 was purified through a heparin-agarose column followed by an ion exchange chromatography. The fraction containing the brown precipitate of [FeS]-IscR (*) was used for EMSA. B. Light scan of the purified IscR protein (from * in panel A). The peak for Fe-S group at 450 nm is indicated by an arrow, and it disappears when the sample was treated with EDTA and DP.

Supplementary Figure S8

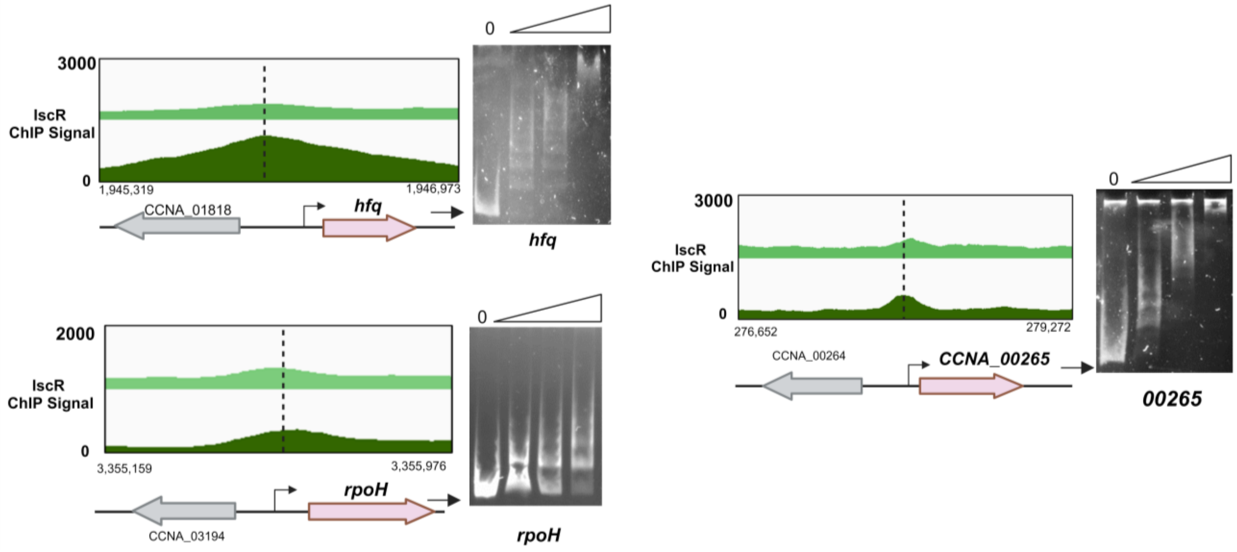

Fig. S8. Selected targets identified by ChIP-Seq bound by IscR. Representative scheme of the peaks obtained for IscR-bound DNA reads in the ChIP-Seq experiment from cultures in PYE medium (light green) or PYE + DP (dark green) visualized by the Integrated Genome Browser. Electrophoresis Mobility Shift Assay (EMSA) experiments using DNA probes corresponding to each peak are shown at the right of the respective scheme. Probes were incubated with increasing concentrations (0-50-100-250 nM) of the purified IscR-His protein, as shown above each figure. Gels were stained with ethidium bromide Created in BioRender. Santos, N. (2024) BioRender.com/h08c318

Supplementary Figure S9

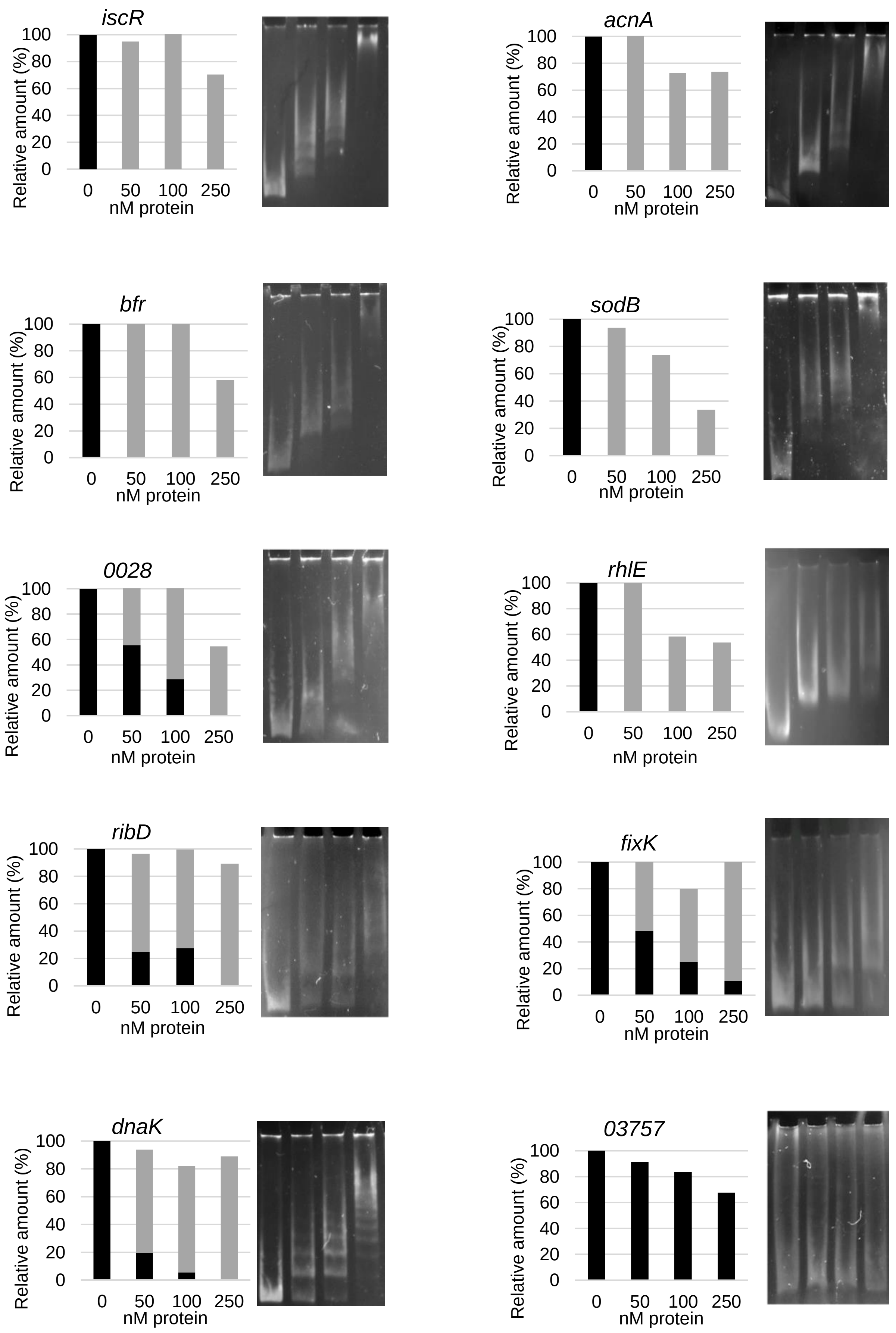

Supplementary Figure S9 (cont)

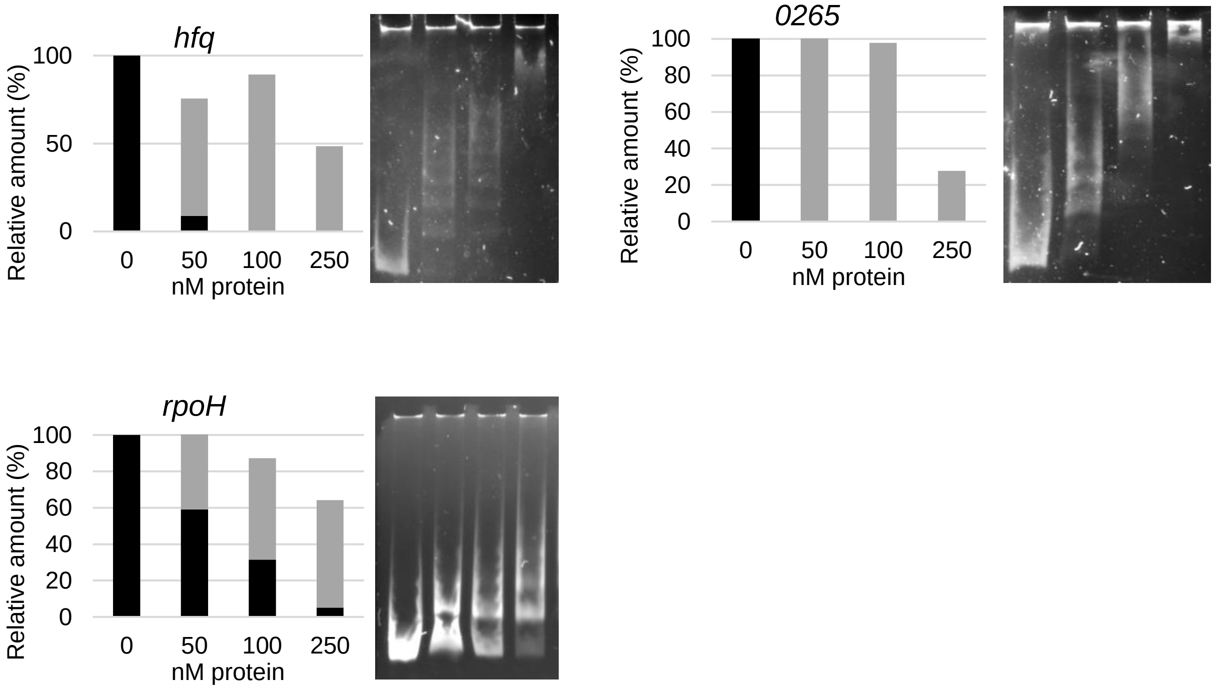

Figure S9. Quantification of the bands from the EMSA. Quantification was carried out with the program ImageJ (https://imagej.net/ij/). The results are shown as relative amounts (% of band intensities relative to the intensity of the initial free DNA band). Black bars indicate the percentage of free DNA, gray bars indicate the percentage of bound DNA. The lower values for some of the bound DNA detected at higher protein concentrations are a result of the difficulty in quantification at the top of the gels due to absence of clear bands.

Supplementary Figure S10

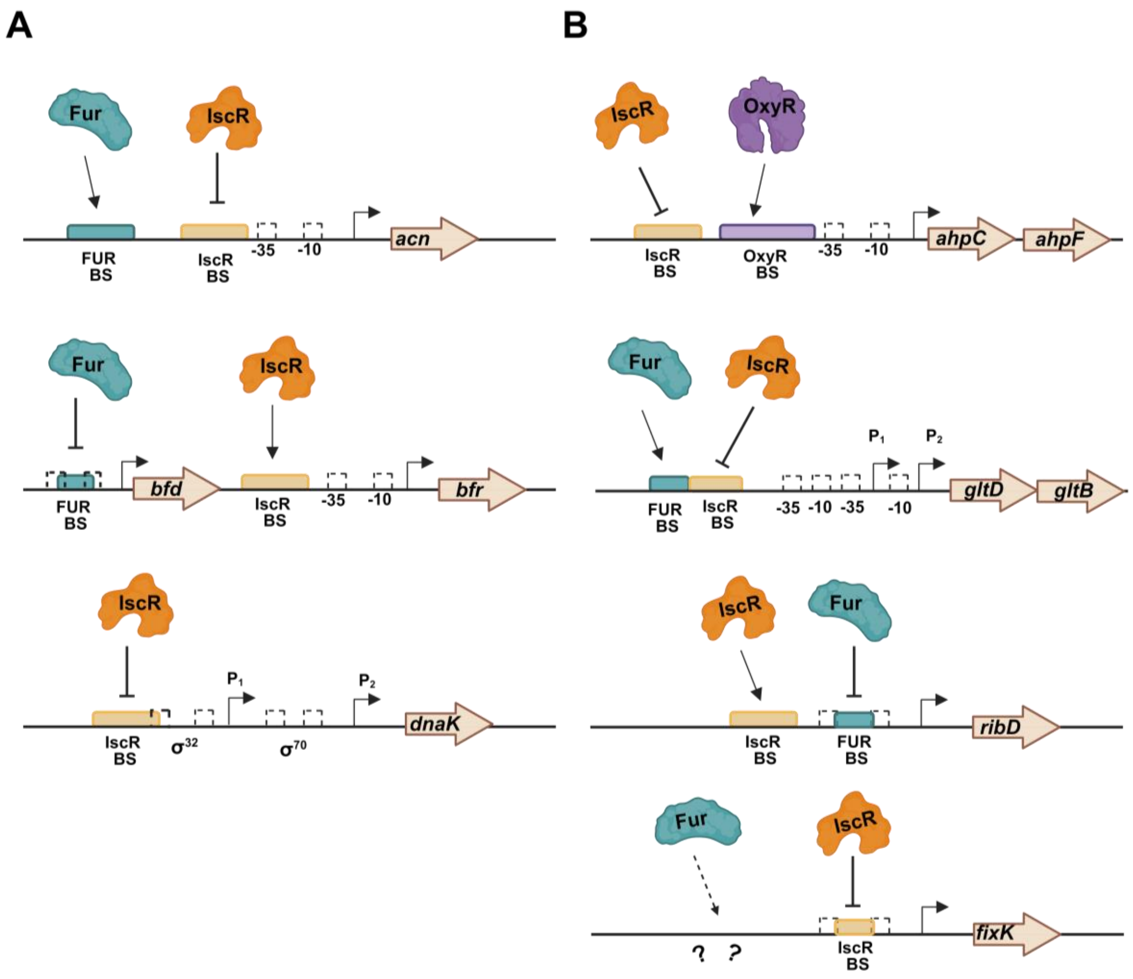

Figure S10. Schematic representation of IscR binding along with other regulators to selected regulatory regions. The regulators and their binding sites are indicated with same colors. Fur binding sites were identified in (da Silva Neto et al., 2009; 2013) and OxyR binding site in (Italiani et al., 2011). Only the first gene is shown in the *rib* operon. The effect of the regulator in gene expression is indicated as activation (arrow heads) or repression (blunt heads). Transcription start sites are indicated by bent arrows. Promoter -35 and -10 regions are indicated by boxes with dotted lines. No Fur binding site was identified in the regulatory region of *fixK* (dotted arrow, ?). Created in BioRender. Santos, N. (2024) BioRender.com/l60p233.

REFERENCES

Larkin MA, G Blackshields, N P Brown, R Chenna, P A McGettigan, H McWilliam, F Valentin, I M Wallace, A Wilm, R Lopez, J D Thompson, T J Gibson, D G Higgins. Clustal W and Clustal X version 2.0. Bioinformatics. 2007 Nov 1;23(21):2947-8. doi: 10.1093/bioinformatics/btm404.

Oberto J. SyntTax: a web server linking synteny to prokaryotic taxonomy. BMC Bioinformatics 14: 4 (2013). doi: 10.1186/1471-2105-14-4.

Sievers F, Wilm A, Dineen DG, Gibson TJ, Karplus K, Li W, Lopez R, McWilliam H, Remmert M, Söding J, Thompson JD, Higgins DG (2011). Fast, scalable generation of high-quality protein multiple sequence alignments using Clustal Omega. Molecular Systems Biology 7:539 doi:10.1038/msb.2011.75

Italiani, V. C. S., Da Silva Neto, J. F., Braz, V. S., and Marques, M. V. (2011). Regulation of catalase-peroxidase KatG is OxyR dependent and fur independent in Caulobacter crescentus. J Bacteriol 193, 1734–1744. doi: 10.1128/JB.01339-10

da Silva Neto, J. F., Braz, V. S., Italiani, V. C. S., and Marques, M. V. (2009). Fur controls iron homeostasis and oxidative stress defense in the oligotrophic alpha-proteobacterium Caulobacter crescentus. Nucleic Acids Res 37, 4812–4825. doi: 10.1093/nar/gkp509

da Silva Neto, J. F., Lourenço, R. F., and Marques, M. V. (2013). Global transcriptional response of Caulobacter crescentus to iron availability. BMC Genomics 14, 1–16. doi: 10.1186/1471-2164-14-549
